# Clinical whole-genome sequencing in severe early-onset epilepsy reveals new genes and improves molecular diagnosis

**DOI:** 10.1093/hmg/ddu030

**Published:** 2014-01-25

**Authors:** Hilary C. Martin, Grace E. Kim, Alistair T. Pagnamenta, Yoshiko Murakami, Gemma L. Carvill, Esther Meyer, Richard R. Copley, Andrew Rimmer, Giulia Barcia, Matthew R. Fleming, Jack Kronengold, Maile R. Brown, Karl A. Hudspith, John Broxholme, Alexander Kanapin, Jean-Baptiste Cazier, Taroh Kinoshita, Rima Nabbout, The WGS500 Consortium, David Bentley, Gil McVean, Sinéad Heavin, Zenobia Zaiwalla, Tony McShane, Heather C. Mefford, Deborah Shears, Helen Stewart, Manju A. Kurian, Ingrid E. Scheffer, Edward Blair, Peter Donnelly, Leonard K. Kaczmarek, Jenny C. Taylor

**Affiliations:** 1 Wellcome Trust Centre for Human Genetics, University of Oxford, Oxford, UK,; 2 Departments of Cellular and Molecular Physiology and Pharmacology, Yale University School of Medicine, New Haven, CT, USA,; 3 NIHR Biomedical Research Centre, Oxford, UK,; 4 Department of Immunoregulation, Research Institute for Microbial Diseases, Osaka University, Osaka, Japan,; 5 Department of Pediatrics, Division of Genetic Medicine, University of Washington, Seattle, WA, USA,; 6 Neurosciences Unit, UCL-Institute of Child Health, London, UK,; 7 Department of Neurology, Great Ormond Street Hospital, London, UK,; 8 Department of Paediatric Neurology, Centre de Reference Epilepsies Rares, Hôpital Necker-Enfants Malades, Paris, France,; 9 Illumina Inc., San Diego, CA, USA,; 10 Departments of Medicine and Paediatrics, Florey Institute, The University of Melbourne, Austin Health and Royal Children's Hospital, Melbourne, VIC, Australia,; 11 Department of Clinical Neurophysiology, John Radcliffe Hospital, Oxford, UK,; 12 Department of Paediatrics, Children's Hospital Oxford, John Radcliffe Hospital, Oxford, UK; 13 Department of Clinical Genetics, Oxford University Hospitals NHS Trust, Oxford, UK

## Abstract

In severe early-onset epilepsy, precise clinical and molecular genetic diagnosis is complex, as many metabolic and electro-physiological processes have been implicated in disease causation. The clinical phenotypes share many features such as complex seizure types and developmental delay. Molecular diagnosis has historically been confined to sequential testing of candidate genes known to be associated with specific sub-phenotypes, but the diagnostic yield of this approach can be low. We conducted whole-genome sequencing (WGS) on six patients with severe early-onset epilepsy who had previously been refractory to molecular diagnosis, and their parents. Four of these patients had a clinical diagnosis of Ohtahara Syndrome (OS) and two patients had severe non-syndromic early-onset epilepsy (NSEOE). In two OS cases, we found *de novo* non-synonymous mutations in the genes *KCNQ2* and *SCN2A.* In a third OS case, WGS revealed paternal isodisomy for chromosome 9, leading to identification of the causal homozygous missense variant in *KCNT1*, which produced a substantial increase in potassium channel current. The fourth OS patient had a recessive mutation in *PIGQ* that led to exon skipping and defective glycophosphatidyl inositol biosynthesis. The two patients with NSEOE had likely pathogenic *de novo* mutations in *CBL* and *CSNK1G1*, respectively. Mutations in these genes were not found among 500 additional individuals with epilepsy. This work reveals two novel genes for OS, *KCNT1* and *PIGQ*. It also uncovers unexpected genetic mechanisms and emphasizes the power of WGS as a clinical tool for making molecular diagnoses, particularly for highly heterogeneous disorders.

## INTRODUCTION

Many recent studies have successfully used whole-exome or whole-genome sequencing (WES, WGS) to uncover the genetic basis of rare disorders (reviewed by 1,2), primarily in a research context. In addition, WES and WGS offer potentially revolutionary approaches to molecular diagnosis for patients in a clinical setting. In order to assess the possible clinical utility of WGS, we have sequenced the genomes of 500 individuals with a variety of medical conditions, including cancer, immunological disease and rare, putatively monogenic syndromes (3–5). As part of this ‘WGS500 project’, we sequenced six patients with severe early-onset epilepsy who had been previously refractory to molecular diagnosis.

Severe early-onset epilepsy is a good candidate for WGS as it is a challenging disorder to understand mechanistically. It represents a broad spectrum of phenotypes which are highly heterogeneous at the clinical and molecular levels (6). While some causative genes have been identified for many of these sub-phenotypes, the limitations of current technologies mean that genetic testing is largely confined to the genes associated with the specific presenting phenotype. However, it is increasingly being recognized that a given gene can cause multiple phenotypes (6), and that more comprehensive genetic testing may improve molecular diagnostic yield (7). This is useful clinically not only because it can help make or confirm a diagnosis, but also because it may allow counseling on recurrence risk and prenatal testing.

In its most severe form, early-onset epilepsy involves frequent seizures beginning in the first three months of life, with abundant epileptic activity that contributes to significant cognitive and motor delay (6,8). It is frequently associated with gross structural brain abnormalities and occasionally with metabolic disorders, which are often genetic in origin (9). Ohtahara Syndrome (OS) is a severe form of early-onset epilepsy characterized by a distinctive electroencephalogram (EEG) pattern known as ‘burst-suppression’, which consists of periodic high voltage bursts of slow waves mixed with spikes, followed by marked attenuation (10). The frequency of OS is about 1 in 100 000 live births (11). Children with OS typically have multiple seizure types including tonic spasms and focal seizures, which are often refractory to anti-epileptic drugs (12). Affected children may progress onto other epilepsy syndromes such as West Syndrome (6), or they may die in infancy.

Several genes have been implicated in severe early-onset epilepsy. The first reported for OS was the X-linked *ARX* gene, which encodes a developmental transcription factor (13). *De novo* mutations in *STXBP1* (14,15), encoding a protein involved in synaptic vesicle release, in *CDKL5* (16), encoding a serine/threonine kinase, and in ion channel genes *KCNQ2* (17), *SCN2A* (18,19) and *SCN8A* (20) have also been implicated, as have recessive mutations in the glutamate transporter *SLC25A22* (21). However, many OS patients test negative for mutations in these genes, indicating that other genes have yet to be identified. Multiple additional genes have been associated with the broader range of early-onset epilepsies, including genes encoding cytoskeletal components [*SPTAN1* (22)] and proteins involved in signaling [*PLCB1* (23)], DNA repair [*PNKP* (24)] and neurotransmitter synthesis [*PNPO* (25)]. However, clinical testing is limited by the availability and costs of conventional single-gene tests, and thus tends to be restricted to genes that have been associated with the specific type of epilepsy. There is therefore scope to apply a more comprehensive approach to diagnosis using whole-genome methods.

In this study, we sequenced six patients with sporadic severe early-onset epilepsy, and their healthy parents. The patients were selected because traditional clinical molecular genetic approaches had failed to uncover the causal mutation. Four of the children had been diagnosed with OS, and two had severe non-syndromic early-onset epilepsy (NSEOE). Because these six cases were all sporadic, and the families were reported as non-consanguineous, we anticipated that the causal mutation was most likely to be *de novo*, but we also considered the simple, compound and X-linked recessive models.

## RESULTS

We sequenced the six trios (Table 1; Supplementary Materials, Note S1) to high coverage on the Illumina HiSeq platform. In searching for the causal mutations, we considered coding variants as well as variants in regulatory regions within 50 kb of known early-onset epilepsy genes (see Materials and Methods). The most plausible causal variant in each trio was a coding mutation, and we report these here. See the Supplementary Materials, Note S2 for a description of candidate variants that were not deemed to be causal.

**Table 1.** Phenotypes and presumed causal mutations in the six trios sequenced

Trio	Phenotype	Age of seizure onset	Current age	Family history	Previous genetic tests	Brain MRI	EEG	Presumed causal mutation	Evidence for pathogenicity
1	OS; severe DD	1 day	5 years	No	arrayCGH, *FRAXA, STXBP1, MECP2, CDKL5, POLG, ARX*	Age 14 days: reduced posterior white matter volume; thin corpus callosum	Age 14 days: Burst suppression	*de novo* in *KCNQ2*: NM_004518:c.C827T: p.T276I	*KCNQ2* previously implicated in OS
2	OS; metopic synostosis; severe DD	1 day	4 years	Paternal great- grandmother and her sister had epilepsy	arrayCGH*, FRAXA*, *MECP2*, *CDKL5*, *STXBP1*	Age 1 year: cerebral atrophy with delayed myelination and hypomyelination	Age 14 days: Burst suppression	Recessive variant in *KCNT1*, homozygous due to UPD9: NM_020822: c.G2896A:p.A966T	*KCNT1* previously implicated in MMPSI and ADNFLE; electrophysiology demonstrated effect on channel current
3	OS; severe DD	14 days	5 years	No	arrayCGH, *CDKL5*, *ARX*, *STXBP1*	Age 8 months: cerebral atrophy, delayed & reduced myelination	Age 6 weeks: Burst suppression	*de novo* in *SCN2A*: NM_001040143: c.A5558G:p.H1853R	*SCN2A* previously implicated in OS
4	OS; severe DD	4 weeks	Deceased age 2 years, 4 months	Mother's cousin died of seizures at age 2	arrayCGH, *MECP2*, *ARX*, *STXBP1*	Age 9 months: delayed and reduced myelination	Age 3 months: Burst suppression	Simple recessive in *PIGQ*: NM_004204:exon3: c.690-2A>G	Binding partner *PIGA* implicated in similar syndrome; mutation leads to exon skipping and reduced GPI synthesis
5	Severe NSEOE; microcephaly; severe DD	2 days	19 years	No	arrayCGH, *MECP2*, *UBE3A*, *TCF4*	Microcephalic (OFC <3rd percentile); structurally normal brain	Age 8 years: multifocal seizure potential on background of significant disruption of cortical function	*de novo* in *CSNK1G1*: NM_022048:c.C688T: p.R230W	*CSNK1G1* involved in synaptic transmission
6	Severe NSEOE; severe DD; PDA and ASD as neonate; cutaneous hypopigmentation	2.5 months	11 years	No	arrayCGH, *MECP2*, *CDKL5, STXBP1*	Cerebral hypoplasia; microcephaly (OFC < 0.4th percentile)	Age 6 years: background diffusely of low amplitude, with multifocal sharp waves	*de novo* in *CBL*: NM_005188:exon9: c.1228-1G>A	*CBL* implicated in NCFC syndrome; mutation leads to exon skipping

OS, Ohtahara syndrome; NSEOE, non-syndromic early-onset epilepsy; DD, developmental delay; PDA, patent ductus arteriosis; ASD, atrial septal defect; OFC, occipital frontal cortex; MMPSI, malignant migrating partial seizures of infancy; ADNFLE, autosomal dominant nocturnal frontal lobe epilepsy; GPI, glycosylphosphatidyl inositol; NCFC, neuro-cardio-facial-cutaneous; UPD, uniparental disomy. More detailed clinical descriptions, including seizure types, head circumference and treatments, are given in Supplementary Materials, Note S1.

### Patients 1 and 3: *KCNQ2* and *SCN2A*

Two OS cases had *de novo* non-synonymous mutations in genes encoding ion channel subunits, *KCNQ2* and *SCN2A* (Table 1; Supplementary Materials, Fig. S1A and B). The *KCNQ2* mutation, NM_004518:c.C827T:p.T276I, falls in the highly conserved fifth transmembrane segment of the channel that forms part of the pore. It is two amino acids away from the T274M mutation recently described in an OS patient (26). The *SCN2A* mutation in Patient 3, NM_001040143:c.A5558G:p.H1853R, is in the cytosolic C-terminal region of the protein. It falls within the 250 residue domain that binds FGF14, which is required for localization at the axon initial segment (27). Other *de novo* mutations in the cytosolic domains were recently reported in patients with OS (18). These reports provide strong supporting evidence that these *de novo* mutations, which have not been previously reported in any epilepsy patients, are responsible for OS in these children.

### Patient 2: *KCNT1*

**Figure 1.**
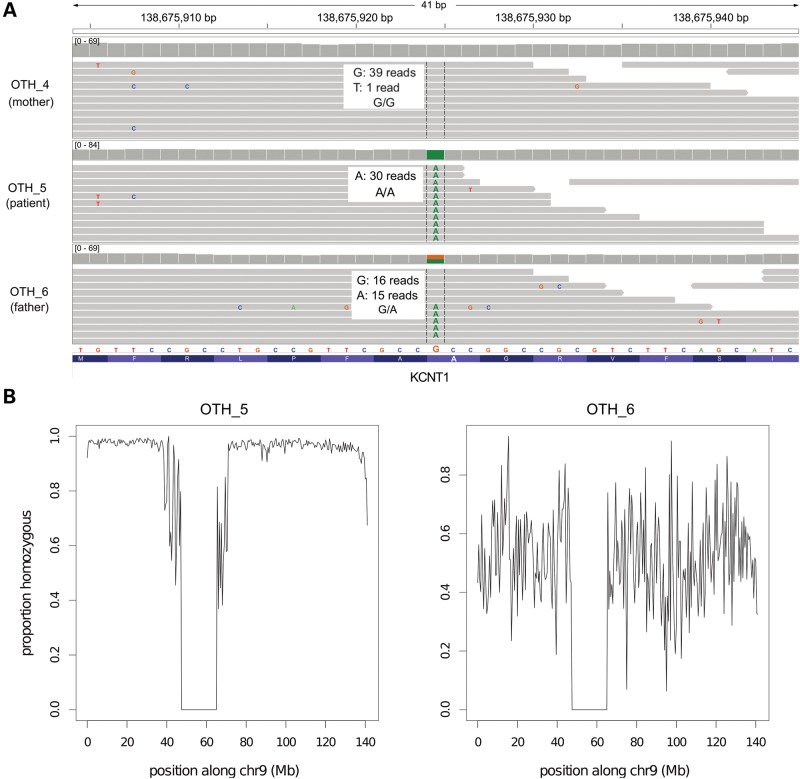
Paternal isodisomy in Patient 2. (**A**) We observed multiple Mendelian errors on chromosome 9 which led us to suspect uniparental disomy (UPD). All variants in the patient (OTH_5) appeared to have been inherited from his father (OTH_6). The *KCNT1* variant is illustrated here as an example. Grey bars represent individual sequencing reads from the sample indicated on the left, and colored letters divergences from the reference sequence. The grey ‘pile-up’ along the top indicates the sequence coverage. The genotype of each individual is shown. (**B**) These plots show the proportion of variants that are homozygous in 500 kb windows across chr9. OTH_5 was completely homozygous, apart from a few spurious calls; the pattern is similar to that seen on chromosome X in males. His father, OTH_6, is shown for comparison. Note that the dip in the middle represents the centromere.

Patient 2 had very severe early-onset epilepsy, an EEG consistent with OS (Supplementary Material, Fig. S2), and profound developmental delay. He had a paternal family history of childhood idiopathic epilepsy affecting his father's maternal aunt, grandmother and nephew. Patient 2 did not have any compelling *de novo* mutations. However, low chromosomal heterozygosity and detection of multiple Mendelian errors (Fig. 1; Supplementary Materials, Fig. S1C) suggested that he had paternal isodisomy for chromosome 9. This was subsequently confirmed by SNP array (Supplementary Materials, Fig. S3; see Supplementary Materials, Note S3). This finding prompted two new alternative hypotheses: that the patient's symptoms were due to aberrant expression of an imprinted gene on chromosome 9, or that there was a recessive pathogenic mutation on this chromosome that had become homozygous as a result of the isodisomy. There was no evidence in the literature for imprinted genes on this chromosome that had plausible links to epilepsy. We therefore scanned the patient's chromosome 9 for rare homozygous variants that might be pathogenic, including around *STXBP1* and *SPTAN1*. The only plausible candidate was a novel non-synonymous variant in *KCNT1* at 9q34.3 that disrupted a highly conserved alanine residue in the intracellular C-terminal domain, NM_020822:c.G2896A:p.A966T. This gene encodes the Na^+^-activated K^+^ channel known as ‘Slack’, which is very widely expressed throughout the brain (28). Dominant mutations in *KCNT1* were recently reported to cause autosomal dominant nocturnal frontal lobe epilepsy (ADNFLE) (29), malignant migrating partial seizures of infancy (MMPSI) (30,31) and infantile spasms (32). Interestingly, one of the MMPSI patients with a *KCNT1* mutation was described as having a ‘subtle’ burst-suppression EEG (30). Distinct from this patient, however, our Patient 2 did not have migrating seizures, and had a clear burst-suppression EEG pattern. Thus, different mutations in *KCNT1* have heterogeneous phenotypic consequences.

**Figure 2.**
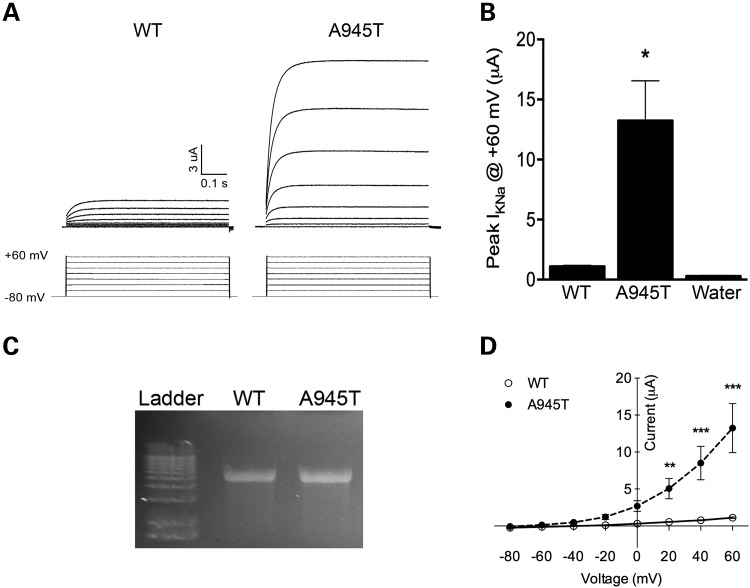
Electrophysiological and channel expression analysis of *KCNT1* mutation found in Patient 2. WT or A945T mutant Slack channel was expressed in *Xenopus laevis* oocytes, and two-electrode voltage clamping (TEVC) performed. (**A**) A representative trace of current activity recorded from an oocyte expressing WT or A945T is shown on top, with the voltage-clamping protocol displayed underneath. (**B**) Averaged quantitation of the peak current is compared at +60 mV (*P* < 0.001, *n* = 5, Student's *t*-test; representative of three independent experiments). (**C**) The quality of RNA injected into *Xenopus* oocytes was checked on a 1% formaldehyde agarose gel. (**D**) Current–voltage relations for the WT or A945T channels. Channel activity as measured at peak current amplitude and normalized to the value at +60 mV is plotted against voltage (**P < 0.01, ***P < 0.001, *n* = 5, two-way ANOVA, Sidak's multiple comparisons test).

The A966 residue in *KCNT1* is completely conserved across all vertebrates for which genome sequences are available. The sequence of the Slack channel C-terminal region is highly conserved between rat and human (92% identical), and residue A966 in the human Slack channel corresponds to residue A945 in the rat protein. We therefore used a rat mutant construct to explore the effect of the novel mutation on channel function, in the same way we described previously for the MMPSI-linked R428Q and A934T mutations (31). We expressed wild-type (WT) and A945T rat Slack in *Xenopus laevis* oocytes, and measured channel activity by performing two-electrode voltage clamping experiments. These experiments showed that activity of the A945T mutant channel was increased significantly by a factor of 13 relative to the WT at +60 mV (Fig. 2A and B). The amounts and integrity of the WT and A945T cRNA used in these experiments appeared similar, just 8% higher in the mutant than the WT (Fig. 2C).

Slack channel activity increases with depolarization (28,33). We therefore compared the voltage dependence of A945T and WT channels. Channel activity of the A945T mutant was significantly greater than that of the WT at all positive potentials (Fig. 2D). However, the voltage dependence of activation of the A945T mutant with depolarization to positive potentials did not differ from that of the WT channel (Fig. 2D) or from that of the previously published reports of Slack channel voltage dependence (31,33). Together, these results suggest that channel opening probability may be greater in the A945T than the WT channel over the same range of depolarized membrane potentials, which would account for the epileptic activity seen in this patient.

### Patient 4: *PIGQ*

Patient 4, who was of West African origin, had severe early-onset epilepsy with a burst-suppression EEG, consistent with OS. Although he was reported to be non-consanguineous, we found several extended homozygous regions in his genome. Within a 2 Mb homozygous region on chr16, he had a novel homozygous single nucleotide variant (SNV) that was predicted to disrupt the highly conserved splice acceptor site of exon 3 of the *PIGQ* gene: NM_004204:exon3:c.690–2A>G. Both parents were heterozygous and two unaffected siblings were either heterozygous or homozygous for the reference allele (Supplementary Materials, Fig. S1D). *PIGQ*, formerly called *GPI1*, encodes a subunit of an *N*-acetylglucosaminyltransferase that catalyzes the first step in glycosylphosphatidyl inositol (GPI) biosynthesis. *PIGQ* stabilizes the enzyme complex (34). A nonsense mutation in the X-linked *PIGA* gene, which encodes another subunit of this enzyme, was recently reported to cause a lethal disorder characterized by multiple congenital abnormalities, structural brain malformations, joint contractures and neonatal seizures with a burst-suppression EEG (35). Recessive mutations in other GPI synthesis genes cause clinically heterogeneous syndromes, all of which involve seizures (36–39). Thus, this *PIGQ* mutation seemed a very plausible candidate for causing OS in Patient 4.

**Figure 3.**
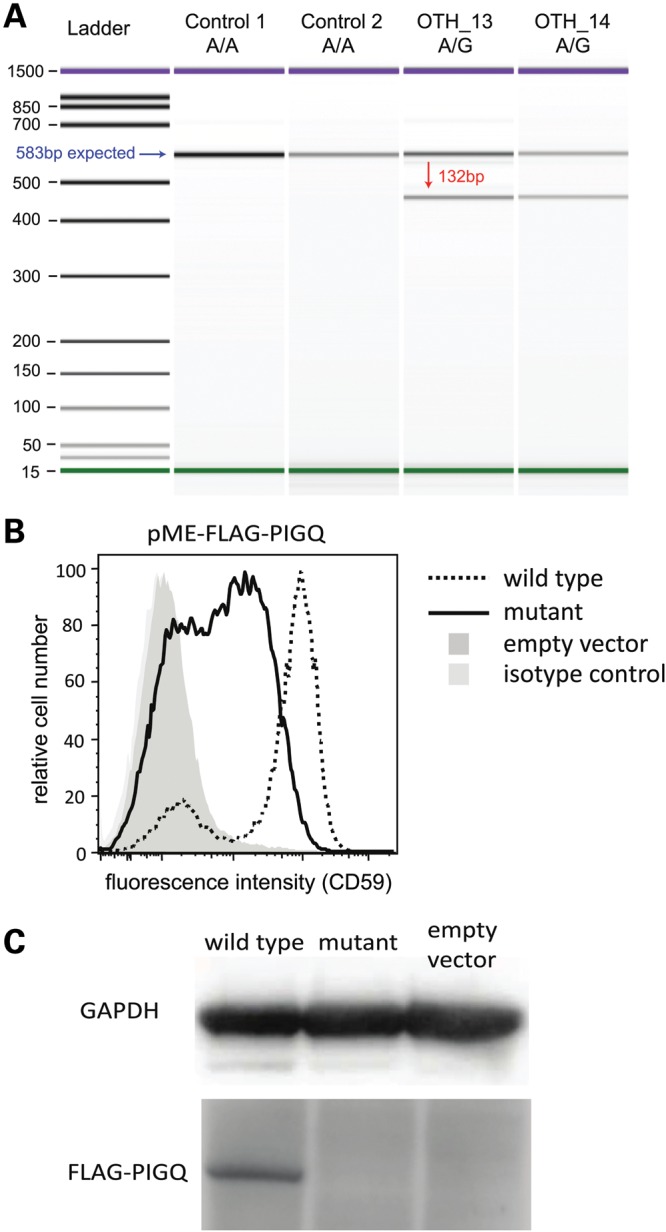
PIGQ splicing mutation in Patient 4. (**A**) The variant causes skipping of exon 3. This image shows the Bioanalyzer gel from an RT-PCR (see Materials and Methods) and demonstrates the presence of two PIGQ transcripts in the heterozygous parents (OTH_13, OTH_14). The blue arrow indicates the band expected from the annotated transcript, and the red arrow that expected from the skipping of exon 3. (**B**) Severely decreased functional activity of the mutant *PIGQ. PIGQ*-deficient CHO cells were transiently transfected with WT or mutant *PIGQ* cDNA (lacking exon 3). Restoration of the surface expression of CD59, a GPI-anchored protein, was assessed by flow cytometry after staining with anti-CD59 antibody. The mutant *PIGQ* did not restore the surface expression of CD59 as efficiently as the WT. X axis, fluorescence intensity corresponding to CD59 expression level per cell; Y axis, relative cell number. (**C**) The expression of mutant protein was greatly decreased and could not be detected by western blotting.

The patient died before we discovered this mutation so we were unable to obtain samples to test the effect of this homozygous mutation on *PIGQ* activity. However, we obtained RNA samples from his parents' blood and examined *PIGQ* splicing. There were two *PIGQ* transcripts, one consistent with the reference annotation and another with a deletion of exon 3, as expected given that the parents were heterozygous for the variant at the splice acceptor site for this exon (Fig. 3A; Supplementary Materials, Fig. S4). Since exon 3 falls immediately before the catalytic domain of *PIGQ*, the mutation seemed likely to abrogate the function of the enzyme and lead to a reduction in GPI synthesis, as was seen for the nonsense mutation in *PIGA* (35). We tested the parents for abnormalities in serum alkaline phosphatase levels and in expression of CD59 on red blood cells, which are typical signs of impaired GPI synthesis (35,36,40), but found none. This is not entirely surprising since heterozygous carriers of other *PIG* gene mutations had normal CD59 levels (37).

The skipping of exon 3 causes an in-frame deletion of 44 amino acids (Fig. 3A). To assess whether this abnormality affected PIGQ function *in vitro*, we transfected human *PIGQ* cDNA either with or without exon 3 into *PIGQ*-deficient Chinese hamster ovary (CHO) cell lines. The mutant *PIGQ* did not restore the surface expression of GPI-anchored proteins (GPI-AP) as efficiently as the WT (Fig. 3B). Additionally, the expression of mutant protein was greatly decreased to a level undetectable by western blotting (Fig. 3C). These results demonstrated that the PIGQ protein lacking the 44 amino acids had some functional activity but was unstable, and so GPI synthesis was impaired. Over 150 proteins have been reported to have GPI anchors (41), including several with important roles in neural development and function (42–44). Further work is needed to determine which of these provides the causal link with epilepsy.

### Patient 5: *CSNK1G1*

Patient 5 had severe tonic-clonic epilepsy, microcephaly and developmental delay. We found a *de novo* non-synonymous mutation at a highly conserved site in *CSNK1G1* (NM_022048:c.C688T:p.R230W; Supplementary Materials, Fig. S1E). This gene encodes casein kinase 1 (CK1), gamma 1, a serine/threonine kinase expressed in many tissues including the brain. CK1 regulates the phosphorylation of NMDA receptors and is thus important for synaptic transmission (45) (see Supplementary Materials, Note S4). The *CSNK1G1* mutation is a prime candidate in this patient, but genetic validation studies did not find any additional patients (see below) and further work is needed to definitively establish it as causal.

### Patient 6: *CBL*

Patient 6 had severe tonic epilepsy, microcephaly and developmental delay. We detected a *de novo* mutation in a highly conserved splice site in the *CBL* gene (NM_005188:exon9:c.1228-1G>A), and showed that this led to exon skipping (Supplementary Materials, Fig. S5). Cbl is a ubiquitously expressed adaptor molecule and ubiquitin ligase that regulates the Ras/MAPK pathway (46). It is primarily recognized as a tumor suppressor (47), but germline mutations in it and other genes involved in the Ras/MAPK pathway can also cause various developmental disorders collectively known as the neuro-cardio-facial-cutaneous (NCFC) syndromes or ‘RAS-opathies’ (48). Notably, mutations in *KRAS* and *BRAF* were recently reported in two boys with refractory epilepsy and cardio-facial-cutaneous (CFC) syndrome (49). It is likely that this splicing mutation ablates Cbl's ubiquitin ligase activity, thereby over-stimulating Ras/MAPK signaling. This may have disrupted neuronal development and led to severe epilepsy (more details in Supplementary Materials, Note S4).

The patient was reviewed by a number of clinical geneticists. Hypopigmented skin lesions and a history of congenital heart disease were noted, but the clinical diagnosis of a NCFC was not considered likely. Review after this molecular finding did not affect the clinical diagnosis and this patient is still thought not to fit phenotypically into this group of disorders. If this gene is confirmed to be causal, this will widen further the phenotype of the Ras-MAPK disorders.

### Mutation screening in other cases

Using a targeted resequencing approach (50), we screened *KCNT1*, *PIGQ*, *CBL* and *CSNK1G1* in a large cohort of epileptic encephalopathy cases from Australia (Supplementary Materials, Table S1). This included two cases of OS, five of early myoclonic encephalopathy [EME; a syndrome which shares features with OS but which is predominantly myoclonic in nature (51)], and thirty-eight of early-onset epileptic encephalopathy (defined as onset within the first three months of life). We also screened these genes in thirteen other cases of OS and EME (Supplementary Materials, Table S2) from a UK cohort, using Sanger sequencing. We looked for coding variants that would fit a recessive model in *KCNT1* and *PIGQ*, or a *de novo* model in *CBL* and *CSNK1G1*, as was observed in our patients, but found none*.* Two of the OS cases have subsequently been attributed to mutations in other genes (50). Thus, our failure to replicate our findings likely reflects further genetic heterogeneity in severe early-onset epilepsy. We also appreciate the relatively small number of patients we have screened who had similar phenotypes to those described here (total sixteen OS, six EME).

## DISCUSSION

WGS heralds promise as a tool for clinical diagnosis of patients with genetic disease. As part of a wide-ranging program to evaluate the clinical utility of WGS (WGS500), we sequenced six patients with severe early-onset epilepsy who had evaded molecular diagnosis by conventional single-gene clinical screening. In doing so, we identified two new genes for OS, as well as two putative genes for severe early-onset epilepsy. This increases the number of known genes for OS from six [*STXBP1* (14)*, ARX* (13)*, CDKL5* (16)*, KCNQ2* (26)*, SCN2A* (18) and *SLC25A22* (52)] to eight (adding *KCNT1* and *PIGQ*). In two cases, we found an unexpected inheritance mechanism: uniparental disomy of chromosome 9 in Patient 2, and simple recessive inheritance due to likely distant consanguinity in Patient 4.

Of particular interest was the discovery that Patient 2 had a pathogenic mutation in *KCNT1* that became homozygous through isodisomy. To date, there have been two published cases of homozygous mutations due to isodisomy causing syndromes involving seizures (53,54), but no examples of such mutations causing severe epilepsy. This is also the first reported case of paternal isodisomy for chromosome 9, and of an apparently recessive mutation in *KCNT1*; recently reported mutations causing MMPSI (31), ADNFLE (29) and infantile spasms (55) were all dominant. Nevertheless, we note that, while this mutation appears to be acting in a recessive manner, there was a paternal history of mild idiopathic epilepsy. It is possible that, in the heterozygous state, this variant predisposes to milder epilepsy, but further testing of affected family members was not possible.

The Slack *KCNT1* A945T mutation had a gain-of-function effect on channel activity. Although the two previously characterized MMPSI-causing mutations, R409Q and A913T, were also gain-of-function alterations, the A945T mutation appears to have a much more profound effect. Whereas channel activity was increased by a factor of 3 at +60 mV in the two MMPSI mutant channels (31), the corresponding increase was 13-fold for the A945T mutation. This observation raises the possibility that the patterns of neuronal firing that produce the distinctive EEG patterns characteristic of these different disorders can vary as a function of *KCNT1* channel activity, and can furthermore influence the nature, severity and onset of the seizures.

The *PIGQ* finding emphasizes the importance of the GPI pathway in brain development and function. This gene was not initially considered as a candidate, since the patient did not have the congenital abnormalities found in children with other *PIG* gene mutations, such as polydactyly. However, it became a very plausible candidate after its binding partner, *PIGA*, was reported to cause a lethal disorder involving neonatal seizures (35). We then demonstrated that the *PIGQ* mutation impairs GPI synthesis in a similar manner (Fig. 3). It remains an open question as to how defective GPI synthesis causes epilepsy, although a number of mechanisms have been suggested, including impaired *Cripto* signaling leading to aberrant forebrain development (44), and disruption of contactin-mediated organization of axonal subdomains at the node of Ranvier (42).

We have also made some unexpected findings about the etiology of other non-syndromic forms of early-onset epilepsy. Although the mutations in patients 5 and 6 have not been definitively established as causal, our results point to several interesting pathways not generally associated with epilepsy. The *de novo* splicing mutation in *CBL* in Patient 6 implies that this patient's condition is actually a ‘RAS-opathy’ (56), which may be consistent with her congenital heart disease. Other genes in the Ras/MAPK pathway have been reported to cause epileptic encephalopathy (49) but *CBL* has not. It has been implicated as a cause of juvenile myelomonocytic leukemia (57,58) and also of a Noonan-like syndrome with microcephaly (57,59). A clinical review after our discovery confirmed that Patient 6 did not have facial features typical of Noonan syndrome. Thus, our results suggest that *CBL* mutations may give rise to an even wider spectrum of phenotypes than previously thought.

Our discovery of the *de novo* non-synonymous *CSNK1G1* mutation in Patient 5 hints that the epileptogenic mechanism may involve the Wnt pathway, which CK1 regulates (60,61), although disruptions in synaptic transmission due to abnormal phosphorylation of NMDA receptors (45) would be a more direct explanation. Intriguingly, the *Drosophila* homolog of *CSNK1G3* was found to suppress seizures in the Na^+^-channel gain-of-function mutant *para^bss1^* (62). Also, a mutation in *PRICKLE1*, which encodes a regulator of the Dishevelled proteins that are intracellular transducers of Wnt signals (63), has been reported to cause progressive myoclonic epilepsy (61). For both *CBL* and *CSNK1G1*, causality can only be definitely established by finding other mutations in patients with similar phenotypes or by extensive functional work in model organisms. Identifying and screening cases similar to Patient 6, rather than those with a more typical NCFC clinical presentation, may increase the chance of finding further patients with *CBL* mutations.

Other groups have already demonstrated the power of WES and WGS in rapidly pinpointing novel genes underlying a rare disease (1,64), particularly in the case of *de novo* inheritance (65). Although all the cases described in this article could probably have been solved by WES, which would have been considerably cheaper, there is emerging evidence to suggest that WES misses clinically relevant mutations because of unequal or incomplete coverage of exons, particularly around the exon boundaries (66). Given that two of our mutations were in splice sites, this was especially relevant. Additionally, the ability to check for pathogenic non-coding mutations in WGS data, as we have done around known early-onset epilepsy genes, is an additional benefit of this approach.

Our study underlines the significant potential of WGS for providing rapid clinical diagnosis of patients with heterogeneous genetic diseases. The patients being investigated here had undergone numerous genetic, biochemical and imaging tests over many years but had been refractory to diagnosis. Using WGS, three of the six patients (Patients 1–3) received a confirmed molecular diagnosis in a clinically relevant timeframe. Conventional molecular testing would not have included these genes. *KCNT1* had not previously been implicated in OS and would not therefore have been tested for this specific phenotype, even though it was described for other severe epilepsy phenotypes after we started this project (29,31). Similarly, *KCNQ2* and *SCN2A* had only been described for benign seizures (67,68) until recent reports of association with the more severe OS phenotype (17,18). These results have already improved the clinical management of these patients' families by providing informed and accurate reproductive risk counseling and the prospect of prenatal diagnosis for future pregnancies.

For the remaining three patients, candidate mutations likely to cause their epilepsy have been identified. The evidence is particularly strong for *PIGQ*, since a homozygous nonsense mutation in *PIGA* causes a similar phenotype (35), and we demonstrated that our mutation was loss of function. Further genetic and functional validation work is required to prove causality definitively, and this remains a challenge in a clinical setting, particularly for rare diseases. Nevertheless, this situation is expected to improve with greater adoption of these technologies and increased sharing of genetic data in public databases.

In conclusion, our results have led to identification of novel genes for severe epilepsy phenotypes and, in addition, demonstrate the clinical utility of WGS as a means of providing comprehensive and rapid molecular diagnosis for patients with mechanistically complex genetic diseases, with concomitant implications for clinical management of these disorders.

## MATERIALS AND METHODS

### Description of patients

The six patients were recruited through the Oxford Clinical Genetics department. A summary of the main clinical features is given in Table 1, along with a list of the genetic tests they had undergone before entering this study (all of which were negative). All patients also had extensive metabolic tests on blood, urine and cerebral spinal fluid, all of which were normal. A more detailed clinical description is given in Supplementary Materials, Note S1.

### Read mapping and variant calling

WGS was conducted on the Illumina HiSeq platform to a coverage of at least 25×. The reads were mapped to the human reference genome (build 37d5) with Stampy (69), and SNVs and small indels were called with an in-house algorithm, Platypus (70). Variants were annotated relative to RefSeq transcripts using ANNOVAR (71) and relative to all Ensembl transcripts using an in-house tool called VariantAnno.

### Variant filtering strategy

To identify *de novo* mutations in the trios, we first screened for variants that were called as homozygous reference in the parents but heterozygous in the child. We then filtered these based on the genotype likelihood ratio (the difference between the log likelihoods for the most likely and the second most likely genotypes), requiring this to be below −5 in all three individuals. This left an average of 126 candidate *de novo* mutations in each trio [about 70 being expected given a mutation rate of 1.18 × 10^−8^ per base pair per generation (72)]. To remove those likely to be due to technical artifacts or incorrect calling of parental genotypes, we removed variants that had been seen before in WGS500, the 1000 Genomes Project or the NHLBI Exome Sequencing Project (ESP). We then prioritized variants that were predicted to alter the protein sequence in any transcript (non-synonymous SNVs, stop loss or gain variants, indels or splice site mutations), particularly those that were highly conserved across the 46 vertebrate species in the UCSC conservation track. There were 0–3 candidate *de novo* coding mutations per trio.

We also considered a simple recessive or compound heterozygous model in all families, as well as an X-linked model where appropriate. For the compound heterozygous model, we required two coding variants in the same gene, one inherited from each heterozygous parent. Since EOEE is extremely rare (frequency ∼1/100 000), we excluded variants with a frequency greater than 0.005 in 1000 Genomes or ESP, or for which there were any homozygotes or hemizygotes or more than five heterozygotes among the other WGS500 samples (*n* = 294), which included no other patients with seizures.

In addition to screening for coding variants, we also looked for variants that might be affecting regulation of known EOEE genes. Specifically, we focused on variants at conserved positions in regulatory regions within 50 kb of the candidate genes *KCNQ2, SCN2A, SCN1A, SPTAN1, SRGAP2, MAGI1, PLCB1, STXBP1, PNPO, PCDH19, GRIN2A, MAPK10, CDKL5, SLC25A22, ERBB4* and *ARX*. A variant was considered conserved if it had a GERP (73) or phyloP (74) score greater than 2 or a phastCons (75) score greater than 0.95, or was in a GERP constrained element (73) or a phastCons constrained element (75). We used the regulatory regions defined by the Ensembl V65 Regulatory Build (http://www.ensembl.org/info/genome/funcgen/regulatory_build.html, last accessed date on February, 2013).

### Variant validation

All putatively causal variants were Sanger-sequenced to confirm the genotypes of the proband and parents.

### Splicing assays

Fresh blood was collected using PAXgene blood RNA tubes (Becton Dickinson, Oxford, UK) and RNA was extracted using the PAXgene Blood RNA kit (Qiagen). cDNA was synthesized using the QuantiTect Reverse Transcription kit (Qiagen) according to the manufacturer's instructions. We carried out a PCR using the FastStart Kit (Roche) and primers as follows: PIGQ-2F, CACGCAGTGAGGTGCTCTT; PIGQ-5R, GGGGACATGAGGTGGATGTA; CBL-8F, GAGATGGGCTCCACATTCC; CBL-11R, GAACTTGGGGCAGATACTGG. To size PCR products accurately, we used ‘on-chip-electrophoresis’ and ran 1 µl on a DNA 1000 v2.3 chip using the 2100 Bioanalyser system (Agilent). We expected WT RT-PCR products of 583 and 698 bp for NM_004204 (*PIGQ*) and NM_005188 (*CBL*), respectively. Sanger sequencing was used to confirm the identity of the aberrant bands.

### *KCNT1* functional work

#### Site-directed mutagenesis and cRNA synthesis

The rat homologue (A945T) of the human *KCNT1* A966T mutation was created using the QuikChange Mutagenesis kit (Stratagene) using the WT *KCNT1* construct in pOX expression vector as the template, and the following primers: 5′-GTTCCGCCTGCCATTTGCTACTGGTCGGGTGTTTAGTA-3′ (forward) and 5′-TACTAAACACCCGACCAGTAGCAAATGGCAGGCGGAAC-3′ (reverse). The resulting construct was sequenced to confirm the presence of mutation. The cDNA construct was then linearized using NotI, and the complementary RNA (cRNA) made using the mMessage mMachine T3 kit (Ambion). The final reaction was purified using MinElute PCR Purification kit (Qiagen), and RNA eluted in nuclease-free water. RNA purity and concentration was checked using a NanoDrop reader. RNA quality was also checked on a 1% formaldehyde agarose gel, and its densitometry quantitated using ImageJ to confirm its concentration. cRNA was stored at −20°C until ready for use.

#### Electrophysiological characterization in *Xenopus laevis* oocytes

All animal procedures were approved by the IACUC at Yale University. Oocytes were prepared as described previously (31). Defolliculated oocytes were injected with 10 ng of RNA encoding WT or A966T, or with sterile water. Oocytes were kept at 18°C, and two-electrode voltage clamping was performed on days 2–5 post-injection, as reported previously (31,76).

### *PIGQ* functional work

We used a previously described human *PIGQ* expression vector (77). The *PIGQ* mutant construct lacking exon 3 was made by site-directed mutagenesis. *PIGQ*-deficient CHO cells (10.2.1) (78) were transiently transfected with FLAG-tagged WT or mutant *PIGQ* cDNA, driven by an SRα promoter (pME FLAG-PIGQ) together with a luciferase expression plasmid for monitoring transfection efficiency. Two days later, to determine the restoration of GPI-AP expression, cells were stained with anti-CD59 (5H8) antibody followed by phycoerythrin-conjugated anti-mouse IgG, and analyzed by a flow cytometer (Cant II; BD Biosciences, Franklin Lakes, NJ, USA) using the Flowjo software (Tommy Digital Inc., Tokyo, Japan). To determine FLAG-tagged *PIGQ* protein levels, lysates of transfected cells were subjected to SDS–PAGE, and western blotting was performed using anti-FLAG antibody (M2, Sigma, St Louis, MO, USA), with anti-GAPDH (6C5, Life Technologies, Carlsbad, CA, USA) as a loading control. Transfection efficiency was determined by luciferase activity using the Luciferase assay kit (Promega, Madison, WI, USA).

### Screening candidate genes in Australian cohort

We sequenced *PIGQ*, *CSNK1G1*, *CBL* and *KCNT1* in a cohort of 500 epileptic encephalopathy patients from Australia. These patients had a variety of different phenotypes, the breakdown of which is shown in Supplementary Materials, Table S1, with an age of onset ranging from 1 day to 25 years.

We used Molecular Inversion Probes (MIPs) to capture all exon and intron/exon boundaries (5 bp flanking) of target genes (Refseq, hg19 build). Detailed methodology is described elsewhere (79). Briefly, pooled MIPs (Supplementary Materials, Table S3) were used to capture target exons from 100 ng of each proband's DNA and target enrichment was performed by PCR using unique reverse primers for each DNA sample. Pooled libraries were subject to massively parallel sequencing using a 101 paired-end protocol on a HiSeq.

We performed raw read processing as described (79), but use a modified analysis pipeline for variant calling. SNV and indel calling and filtering was performed using the Genome Analysis Tool Kit (GATK version 2.2) (http://www.broadinstitute.org/gatk/, last accessed date on February, 2013). We excluded from further analysis any variants with allele balance >0.70, QUAL < 30, QD < 5 or coverage < 25×, and variants in clusters (window size 10 bp) or in homopolymer runs (5 bp). Variants were annotated with SeattleSeq (version 134; http://snp.gs.washington.edu/SeattleSeqAnnotation134/) and the Exome Sequencing Project dataset (see http://eversusgs.washington.edu/EVS/, last accessed date on February, 2013) used to assess variant frequency in the control population. For dominant (or *de novo*) models, we considered only variants not present in this control sample set. For recessive candidates, we considered variants with a frequency in controls of <1% (European American control frequency). Only non-synonymous, splice-site or frameshift variants were assessed further.

Where family members were available, segregation analysis was performed using a ‘MIP-pick’ strategy. We selected and re-pooled only the MIPs that captured the genomic sequence harboring the rare variant of interest and performed target enrichment PCR and sequencing as above for all relevant probands and family members.

### Screening candidate genes in UK cases

We carried out Sanger sequencing of *CBL*, *CSNK1G1*, *PIGQ* and *KCNT1* in 11 patients with Ohtahara syndrome. Clinical details are given in Supplementary Materials, Table S2. Genomic DNA from participating individuals was extracted from peripheral lymphocytes by standard techniques. All participants gave written informed consent and the study was performed in accordance with the Declaration of Helsinki.

Primer pairs were designed for all coding exons with primer3 software (1,2) (http://bioinfo.ut.ee/primer3/, last accessed date on February, 2013) (Supplementary Materials, Table S4). The exons were amplified by PCR using BioMix™ Red (Bioline Ltd). Two different PCR conditions were carried out to amplify exons: (i) an initial denaturation of 95°C for 5 min, followed by 35 cycles of 45 s denaturation at 95°C, 45 s annealing at 58–62°C (depending on fragment) and 1 min extension at 72°C with a final extension at 72°C for 5 min, or (ii) a touchdown PCR program: an initial denaturation of 95°C for 5 min, followed by 24 cycles of 30 s denaturation at 95°C, 30 s annealing at 62°C (minus 0.5°C per cycle) and 1 min extension at 72°C, followed by 15 cycles of 30 s denaturation at 95°C, 30 s annealing at 50°C and 1 min extension at 72°C with a final extension at 72°C for 10 min. If PCR condition 1 was not successful, PCR condition 2 was applied. PCR products were cleaned up with MicroCLEAN (Web Scientific) and were directly sequenced by the Big Dye Terminator Cycle Sequencing System (Applied Biosystems Inc.). Sequencing reactions were run on an ABI PRISM 3730 DNA Analyzer (Applied Biosystems Inc.) and analyzed using Chromas (http://www.technelysium.com.au/chromas.html, last accessed date on February, 2013).

## AUTHOR CONTRIBUTIONS

H.C.M. analyzed the WGS data. L.K.K. directed the *KCNT1* electrophysiology experiments, which were performed by G.E.K., M.F., M.R.B. and J.K. A.T.P. and K.A.H. did the Sanger sequencing and splicing assays. G.B. made the *KCNT1* construct for electrophysiology, supervised by R.N. J.B., A.K. and J.-B.C. created the bioinformatics infrastructure of the WGS500 project and provided NGS data processing, preparing the BAM files and variant calls. R.C. and A.R. contributed to WGS analysis. Y.M. and T.K. did the *in vitro PIGQ* experiments. S.H. and I.E.S. studied patients in the Australian cohort and performed phenotyping analysis of the larger epilepsy panel, on which G.C. and H.M. performed the MIP sequencing. M.A.K. and E.M. performed phenotyping and Sanger sequencing on the UK cohort. H.S., D.S. and E.B. contributed samples and clinical data from the affected individuals and assisted with the interpretation of results. Z.Z. and T.M. provided clinical data and advice on the phenotypes. E.B., D.B., G.M., J.C.T. and P.D. conceived the study, and L.K.K., E.B., P.D. and J.T. directed it. H.C.M., G.E.K., A.T.P., E.B., I.E.S., L.K.K., J.C.T. and P.D. wrote the article. This project was carried out as part of the WGS500 Consortium.

## SUPPLEMENTARY MATERIAL

Supplementary Material is available at *HMG* online.

## FUNDING

This work was supported in part by a Wellcome Trust Core Award (090532/Z/09/Z) to the Wellcome Trust Centre for Human Genetics and a Wellcome Trust Senior Investigator Award to P.D. (095552/2/11/2), in part by NIH grant HD067517 and in part by the Oxford NIHR Biomedical Research Centre. Funding to pay the Open Access publication charges for this article was provided by the wellcome Trust and the Oxford NIHR Biomedical Research Centre.

## Supplementary Material

Supplementary Data
