# Supplementary material for: Clinical whole-genome sequencing in severe early-onset epilepsy reveals new genes and improves molecular diagnosis
Source: Hum Mol Genet. 2014 Jan 25;23(12):3200–11. doi: 10.1093/hmg/ddu030 (PMC4030775; doi:10.1093/hmg/ddu030)
Supplement: Supplementary Data [file supp_23_12_3200__index.html]

Clinical whole-genome sequencing in severe early-onset epilepsy reveals new genes and improves molecular diagnosis — Clinical whole-genome sequencing in severe early-onset epilepsy reveals new genes and improves molecular diagnosis — Supplementary Data 

# Clinical whole-genome sequencing in severe early-onset epilepsy reveals new genes and improves molecular diagnosis

## Supplementary Data

Supplementary Data

**Files in this Data Supplement:**

- Supplementary Figure 1 - pdf file
- Supplementary Figure 3 - pdf file
- Supplementary Figure 4 - pdf file
- Supplementary Figure 5 - pdf file
- Supplementary Table 4 - pdf file
- Supplementary Data - Doc file
- Supplementary Figure Legends - doc file
- Supplementary Figure 2 - png file
- Supplementary Tables - doc file
- Supplementary Table 3 - xls file
