# Supplementary figures and images for: Clinical whole-genome sequencing in severe early-onset epilepsy reveals new genes and improves molecular diagnosis
Source: Hum Mol Genet. 2014 Jan 25;23(12):3200–11. doi: 10.1093/hmg/ddu030 (PMC4030775; doi:10.1093/hmg/ddu030)

# Figure S1

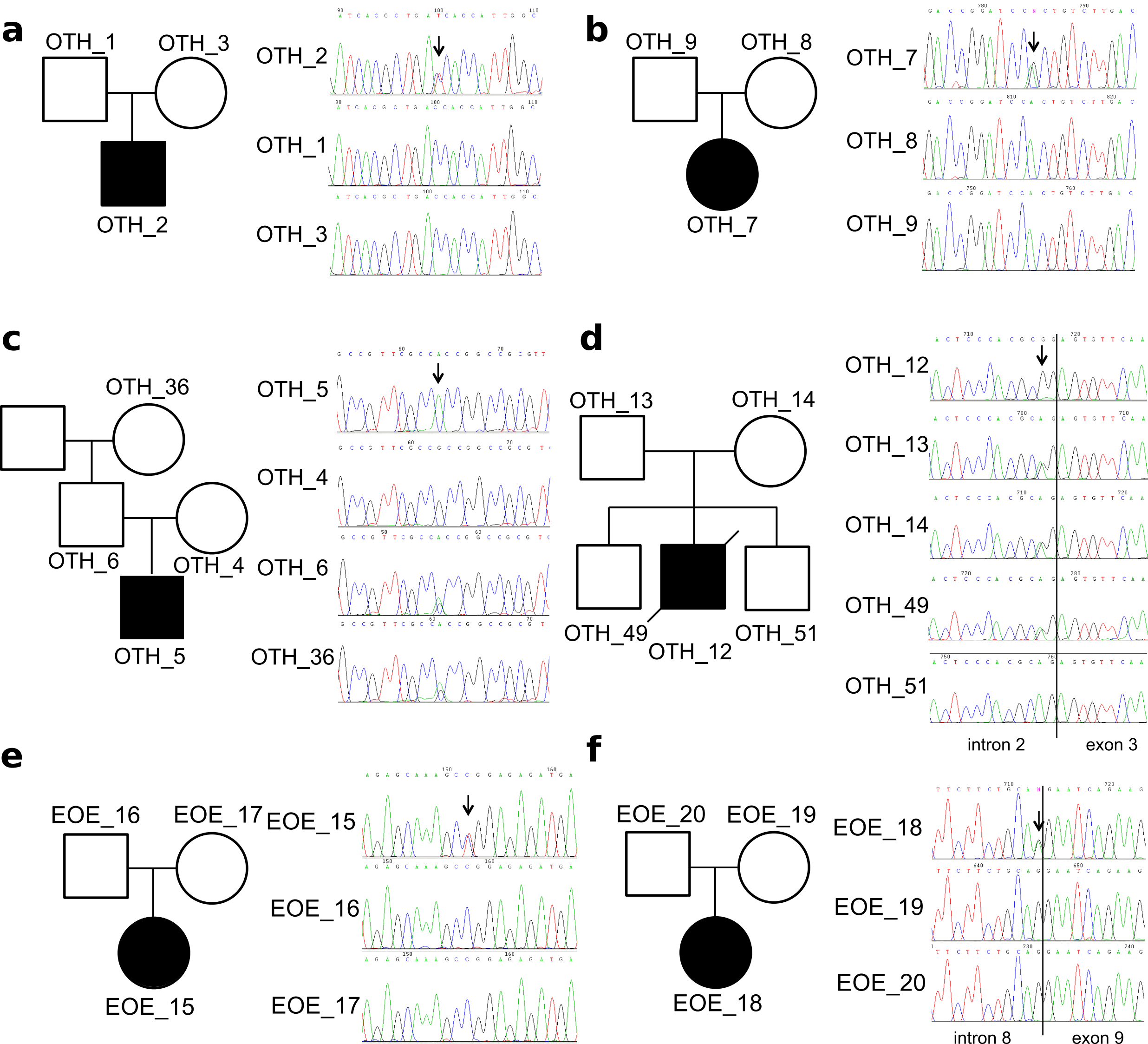

Supplement: Supplementary Data [file supp_ddu030_ddu030supp_fig1.pdf]

# Figure S2

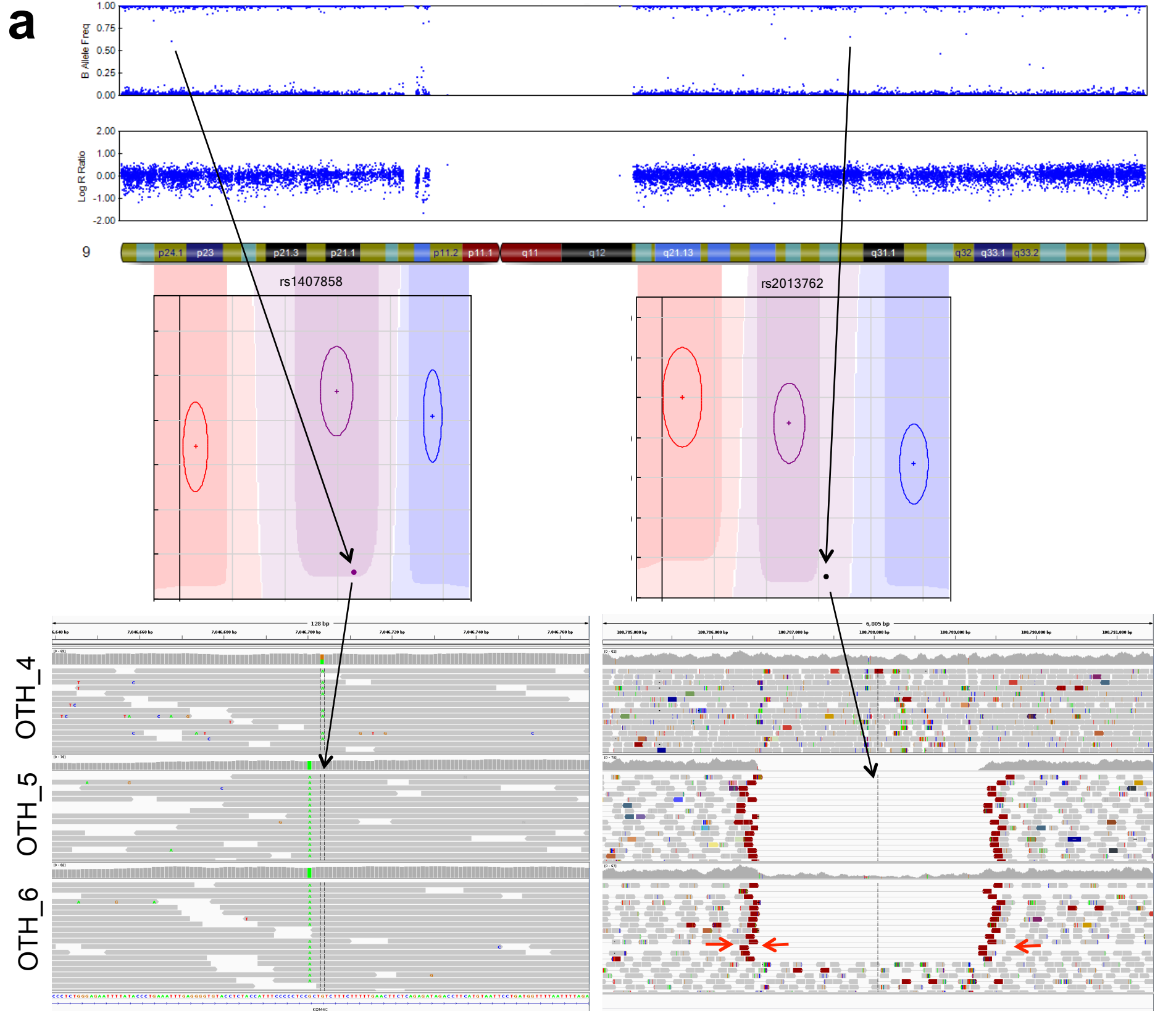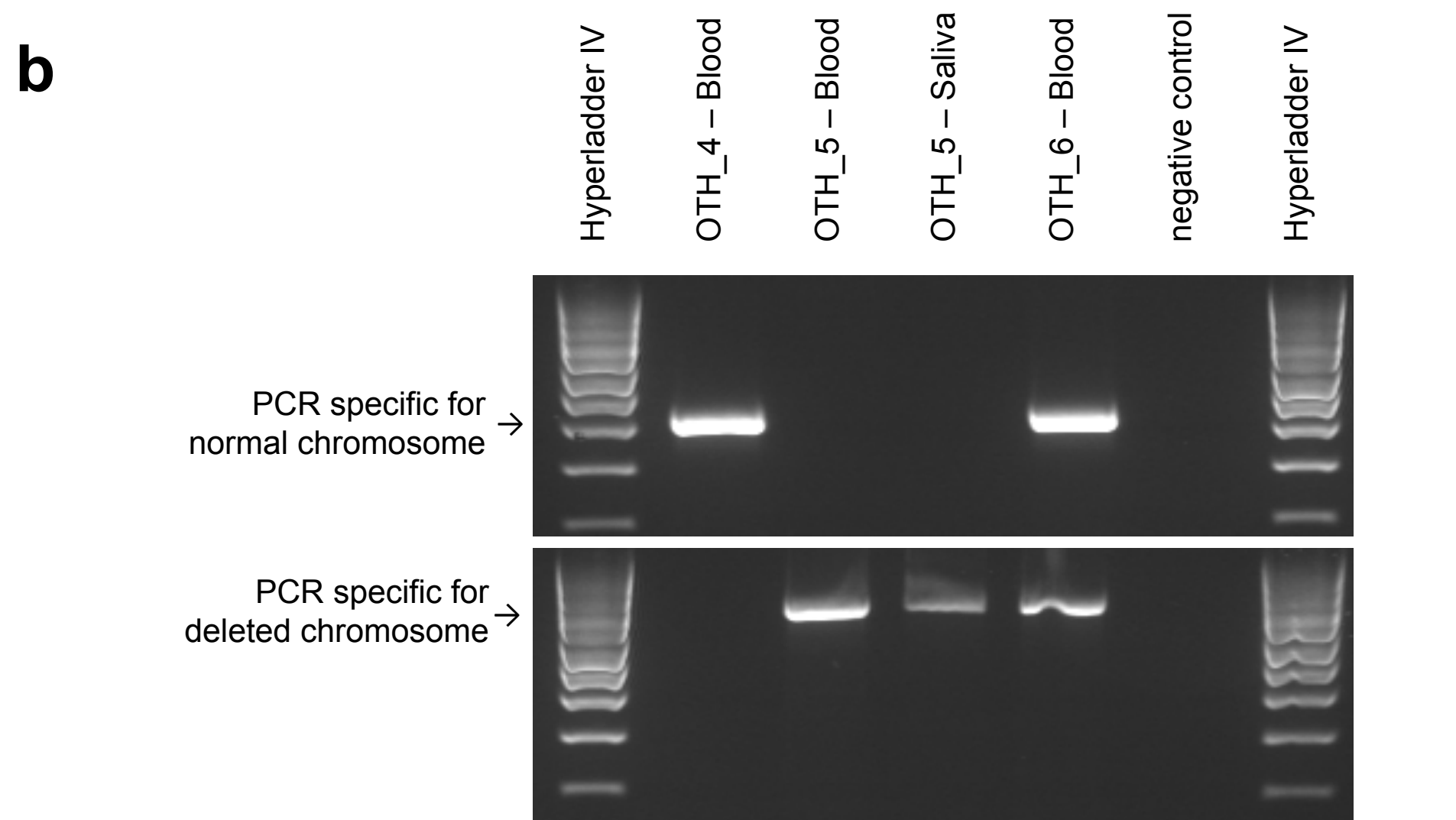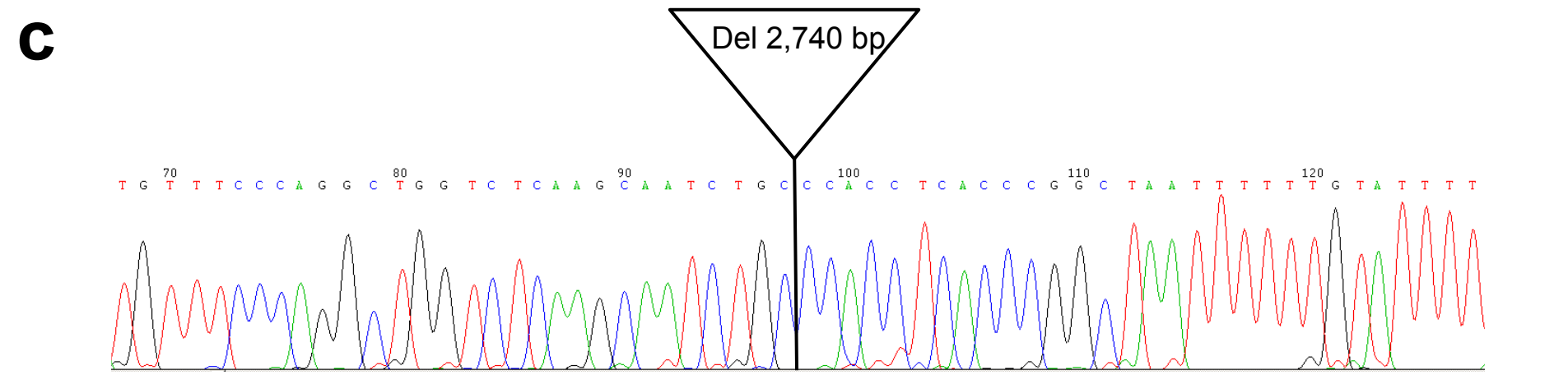

Supplement: Supplementary Data [file supp_ddu030_ddu030supp_fig3.pdf]

# Figure S4

**a**

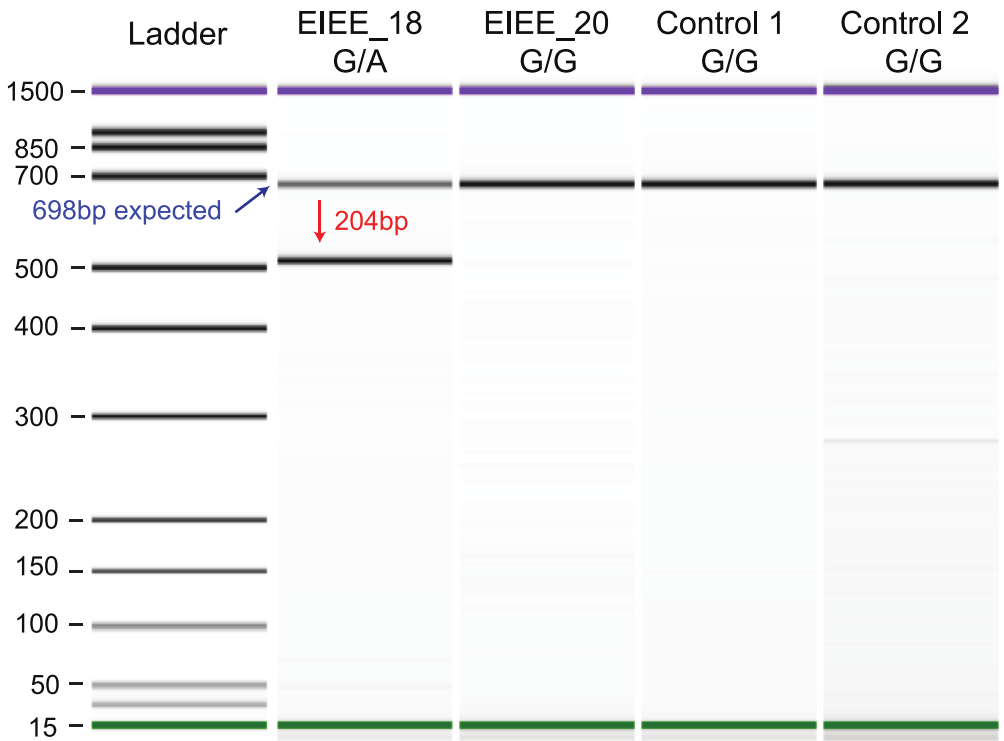

**b**

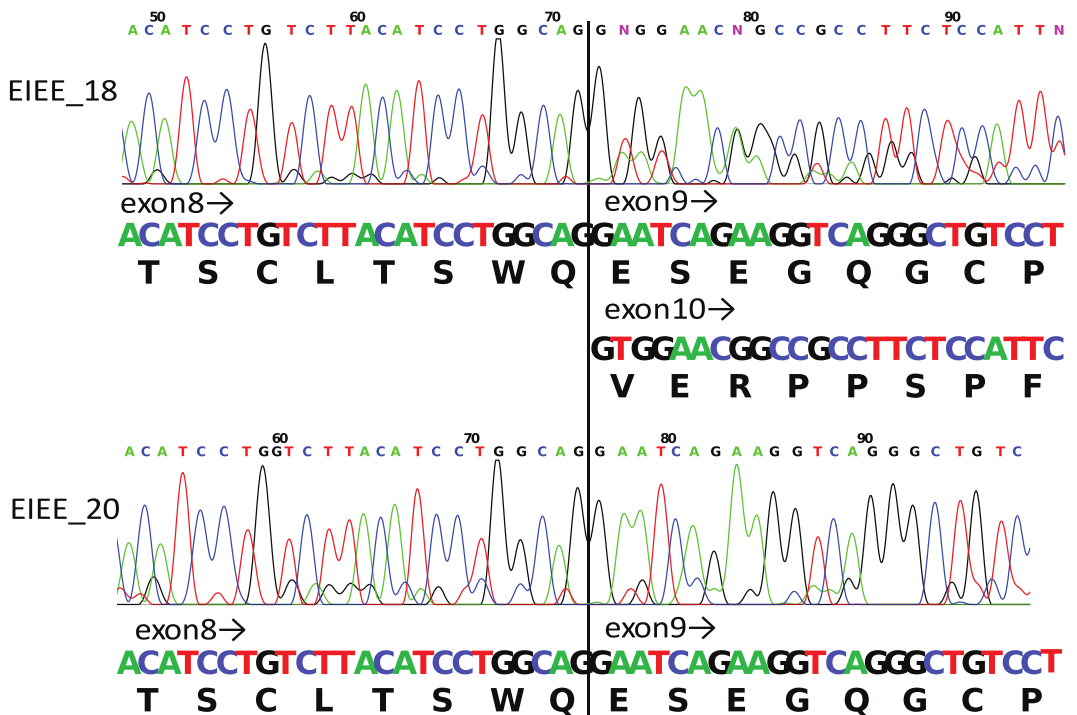

Supplement: Supplementary Data [file supp_ddu030_ddu030supp_fig5.pdf]

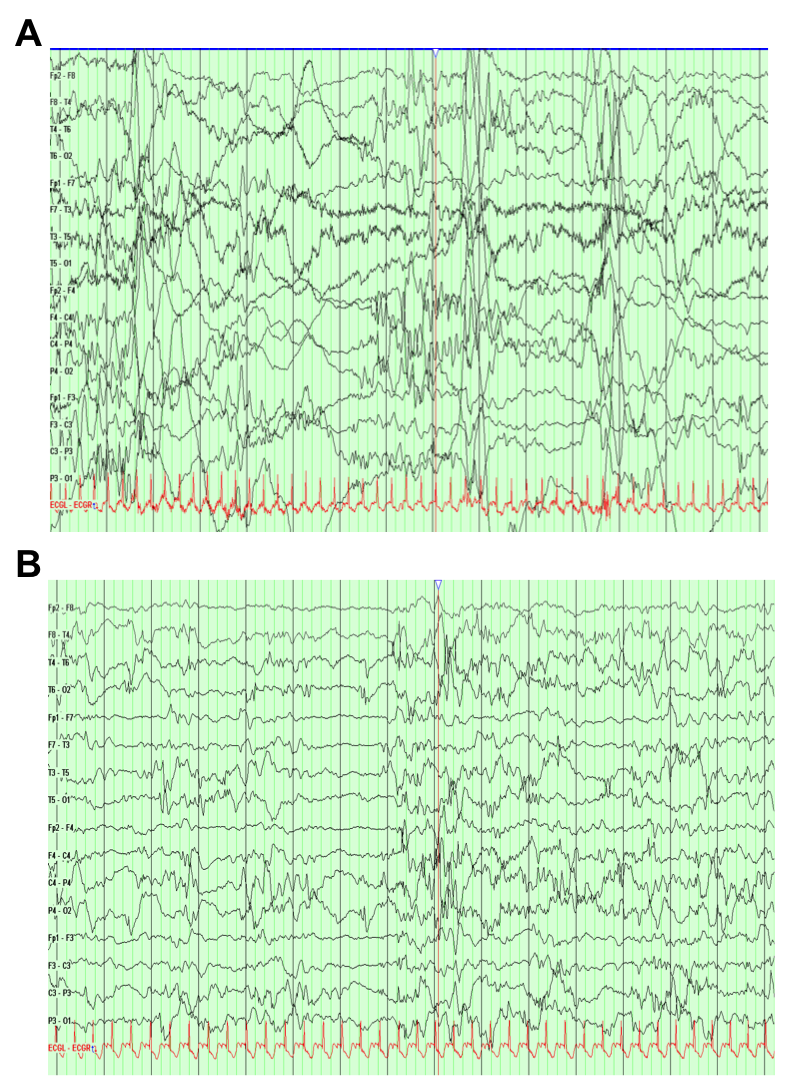

Supplement: Supplementary Data [file supp_ddu030_ddu030supp_fig2.png]
