# Supplementary material for: Clinical whole-genome sequencing in severe early-onset epilepsy reveals new genes and improves molecular diagnosis
Source: Hum Mol Genet. 2014 Jan 25;23(12):3200–11. doi: 10.1093/hmg/ddu030 (PMC4030775; doi:10.1093/hmg/ddu030)
Supplement: Supplementary Data [file supp_ddu030_ddu030supp_fig4.pdf]

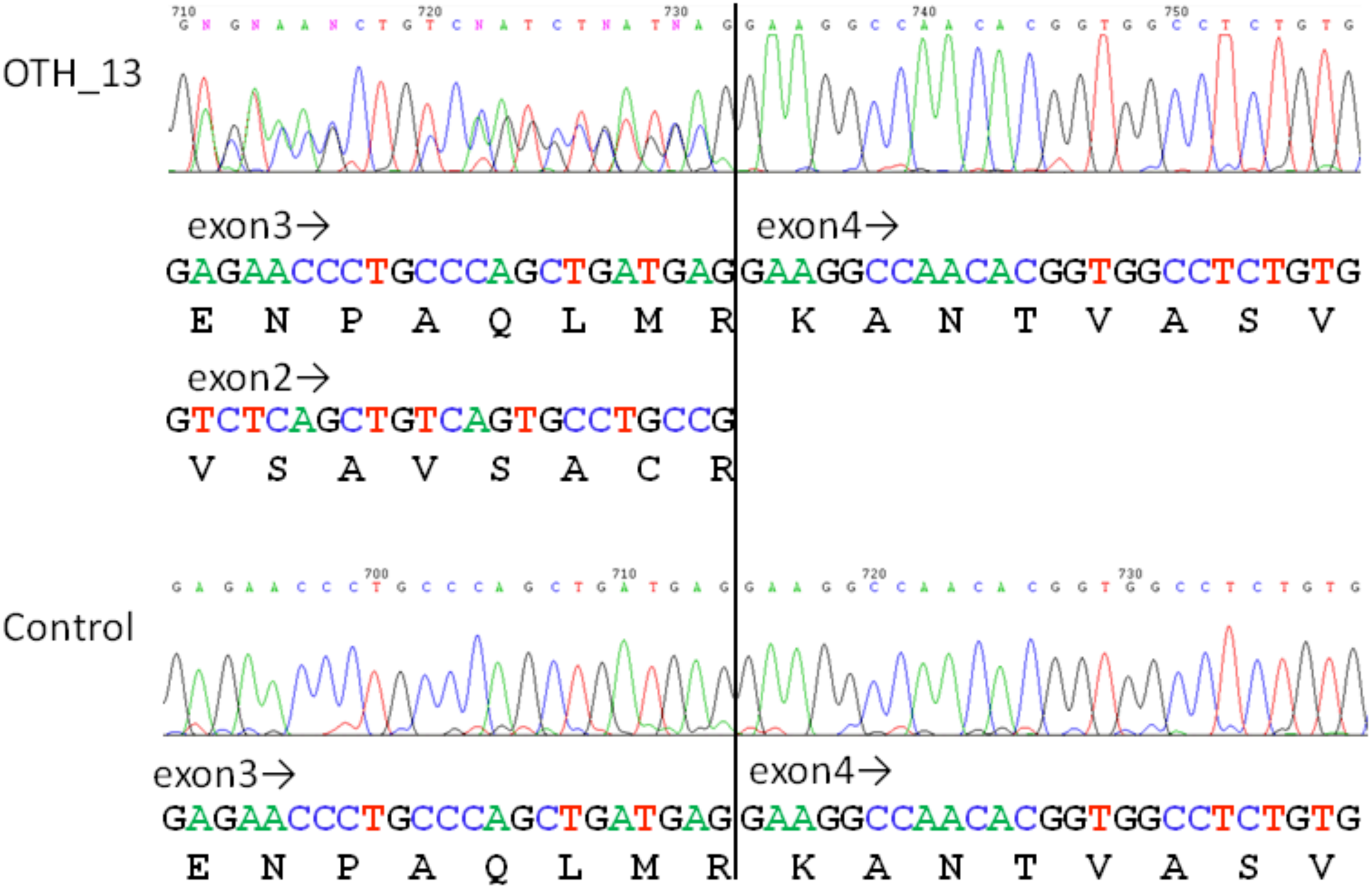
OTH_13
exon3→
GAGAACCCTGCCCAGCTGATGAG
E N P A Q L M R
exon4→
GAAGGCCAACACGGTGGCCTCTGTG
K A N T V A S V
exon2→
GTCTCAGCTGTCAGTGCCTGCCG
V S A V S A C R
Control
exon3→
GAGAACCCTGCCCAGCTGATGAG
E N P A Q L M R
exon4→
GAAGGCCAACACGGTGGCCTCTGTG
K A N T V A S V
