# Supplementary material for: Clinical whole-genome sequencing in severe early-onset epilepsy reveals new genes and improves molecular diagnosis
Source: Hum Mol Genet. 2014 Jan 25;23(12):3200–11. doi: 10.1093/hmg/ddu030 (PMC4030775; doi:10.1093/hmg/ddu030)
Supplement: Supplementary Data [file supp_ddu030_ddu030supp_table4.pdf]

Supplementary Table 4

Name	Sequence (5' → 3')	Amplicon size (bp)	Target transcript
KCNT1-1F	aacgcgagggaagaaggt	280	KCNT1-202 (ENST00000298480)
KCNT1-1R	ggaacttgggggtcttaggt		KCNT1-202 (ENST00000298480)
KCNT1-1bF*	cgggtgcttgaaccttctc	499	KCNT1-201 (ENST00000263604)
KCNT1-1bR*	gtgggcttaggaggggagac		KCNT1-201 (ENST00000263604)
KCNT1-2F	gacgcgggctgaggg	341	KCNT1-202 (ENST00000298480)
KCNT1-2R	caccccctccgaaacttct		KCNT1-202 (ENST00000298480)
KCNT1-2bF*	ccatccgtccgtctatctgt	191	KCNT1-201 (ENST00000263604)
KCNT1-2bR*	gctctcacacctctaagagc		KCNT1-201 (ENST00000263604)
KCNT1-3F	cagttggaaagttggaagaagtc	404	KCNT1-202 (ENST00000298480)
KCNT1-3R	gtctcagacagtgtgaagctaag		KCNT1-202 (ENST00000298480)
KCNT1-4F	cagagagcccagccagac	346	KCNT1-202 (ENST00000298480)
KCNT1-4R	ctttttccatcctgggacag		KCNT1-202 (ENST00000298480)
KCNT1-5F	cttgtctcccaggtgttagagct	575	KCNT1-202 (ENST00000298480)
KCNT1-5R	aactggctgggaactcacaat		KCNT1-202 (ENST00000298480)
KCNT1-6F	agtggaggccaatggttatg	399	KCNT1-202 (ENST00000298480)
KCNT1-6R	gctcactgagttggagtcacc		KCNT1-202 (ENST00000298480)
KCNT1-7F	ctggctttgtgtggtacctg	242	KCNT1-202 (ENST00000298480)
KCNT1-7R	gagagggtgggggaggtg		KCNT1-202 (ENST00000298480)
KCNT1-8/9F	acttcccagcctcatccac	475	KCNT1-202 (ENST00000298480)
KCNT1-8/9R	cggtcctctacaggtcatgc		KCNT1-202 (ENST00000298480)
KCNT1-10F	cgtctgctttccactgtcct	291	KCNT1-202 (ENST00000298480)
KCNT1-10R	ccgatagcttggacataggc		KCNT1-202 (ENST00000298480)
KCNT1-11F	ctggtgatgcacagctcagt	378	KCNT1-202 (ENST00000298480)
KCNT1-11R	agtggcaccctccctctact		KCNT1-202 (ENST00000298480)
KCNT1-12F	ggcccctaacagacagtcg	400	KCNT1-202 (ENST00000298480)
KCNT1-12R	gaggcatctgccctcact		KCNT1-202 (ENST00000298480)
KCNT1-13F	gtgggcacaaacacagtcc	287	KCNT1-202 (ENST00000298480)
KCNT1-13R	tgagcagccgacctgact		KCNT1-202 (ENST00000298480)
KCNT1-14F	ctccacccacgctcag	179	KCNT1-202 (ENST00000298480)
KCNT1-14R	ggcaggtctcttccctcag		KCNT1-202 (ENST00000298480)
KCNT1-15F	ccctgagggaagagacctg	309	KCNT1-202 (ENST00000298480)
KCNT1-15R	ccgtcacacatgtgcacatg		KCNT1-202 (ENST00000298480)
KCNT1-16F	gcaccttcaggaagtgagca	295	KCNT1-202 (ENST00000298480)
KCNT1-16R	ctcctctgggaagcccatc		KCNT1-202 (ENST00000298480)
KCNT1-17F	ggcaaaagtcctgcataggg	338	KCNT1-202 (ENST00000298480)
KCNT1-17R	gagggcacagagcagagc		KCNT1-202 (ENST00000298480)
KCNT1-18F	gtggggactctggtgatttg	605	KCNT1-202 (ENST00000298480)
KCNT1-18R	gcataggccaagaggaactg		KCNT1-202 (ENST00000298480)
KCNT1-19F	tcacctgagctctgggaact	394	KCNT1-202 (ENST00000298480)
KCNT1-19R	accaaggatgctccgacac		KCNT1-202 (ENST00000298480)
KCNT1-20F	agagcagacccagccacct	240	KCNT1-202 (ENST00000298480)
KCNT1-20R	gagaaaccagggcaggtatg		KCNT1-202 (ENST00000298480)

KCNT1-21F	gtggccagcaggaaccac	288	KCNT1-202 (ENST00000298480)
KCNT1-21R	ctggctgcaggtctgagg		KCNT1-202 (ENST00000298480)
KCNT1-22F	ctctgtgcatggagggtagg	234	KCNT1-202 (ENST00000298480)
KCNT1-22R	caccccagactttggaagag		KCNT1-202 (ENST00000298480)
KCNT1-23F	gtcctgtgggtggggagt	238	KCNT1-202 (ENST00000298480)
KCNT1-23R	ggctgtggggtggttta		KCNT1-202 (ENST00000298480)
KCNT1-24F	cccaccctgagacctccta	235	KCNT1-202 (ENST00000298480)
KCNT1-24R	ccctttctcccactctttctg		KCNT1-202 (ENST00000298480)
KCNT1-25F	tgtacggtgcacacacagt	296	KCNT1-202 (ENST00000298480)
KCNT1-25R	ggagtcctatcattcagaac		KCNT1-202 (ENST00000298480)
KCNT1-26F	gtgcctcactgtggctc	246	KCNT1-202 (ENST00000298480)
KCNT1-26R	agagcctcctggccaccgtgaatca		KCNT1-202 (ENST00000298480)
KCNT1-27F	agccaactcagggttcc	307	KCNT1-202 (ENST00000298480)
KCNT1-27R	atctagcccaggtccctgac		KCNT1-202 (ENST00000298480)
KCNT1-28F	ctaagcatgttccgtgcaga	189	KCNT1-202 (ENST00000298480)
KCNT1-28R	ctggcaggcttctccatgt		KCNT1-202 (ENST00000298480)
KCNT1-29F	gagcccacacctcttcctaa	494	KCNT1-202 (ENST00000298480)
KCNT1-29R	ccgactgttcttggctgtg		KCNT1-202 (ENST00000298480)
KCNT1-30F	gcacctcgcttgctgatatt	241	KCNT1-202 (ENST00000298480)
KCNT1-30R	ctcacagctcctgtccacct		KCNT1-202 (ENST00000298480)
KCNT1-31F	atgaggtgctggagcagaat	299	KCNT1-202 (ENST00000298480)
KCNT1-31R	gtgcctgtggcctcatct		KCNT1-202 (ENST00000298480)
KCNT1-32F	gggcactggggagatgag	293	KCNT1-202 (ENST00000298480)
KCNT1-32R	ctcctccacgtccttcctg		KCNT1-202 (ENST00000298480)
PIGQ-2.1F	gaacttgaagctgaggtgcc	572	PIGQ-001 (ENST00000321878)
PIGQ-2.1R	gggcaggtgtagctgtga		PIGQ-001 (ENST00000321878)
PIGQ-2.2F	acgttctggagctgcgag	457	PIGQ-001 (ENST00000321878)
PIGQ-2.2R	cactgcagacaaagggatgg		PIGQ-001 (ENST00000321878)
PIGQ-3/4F	gacggtgggaggaggatct	579	PIGQ-001 (ENST00000321878)
PIGQ-3/4R	gtagaactggctttgtgggg		PIGQ-001 (ENST00000321878)
PIGQ-5F	cctggggagatgcaggtg	249	PIGQ-001 (ENST00000321878)
PIGQ-5R	cctaccctcttcagcaagct		PIGQ-001 (ENST00000321878)
PIGQ-6/7F	cacctcatgtcctgtgtgtg	595	PIGQ-001 (ENST00000321878)
PIGQ-6/7R	tcagctgcaggacgggag		PIGQ-001 (ENST00000321878)
PIGQ-8F	gactgcagctccgggatg	228	PIGQ-001 (ENST00000321878)
PIGQ-8R	cagaagccacacagagcac		PIGQ-001 (ENST00000321878)
PIGQ-9F	caggatggggtcggactg	243	PIGQ-001 (ENST00000321878)
PIGQ-9R	caggtgtggttcccctcc		PIGQ-001 (ENST00000321878)
PIGQ-10F	ggaaggtacctgcagcctc	221	PIGQ-001 (ENST00000321878)
PIGQ-10R	cagacactacacccagctga		PIGQ-001 (ENST00000321878)
PIGQ-10/11.1F	tctctgcagacggggttg	500	PIGQ-001 (ENST00000321878)
PIGQ-10/11.1R	cctggggttgctgtgctc		PIGQ-001 (ENST00000321878)
PIGQ-10/11.2F	ggagtgagcagatgtgggg	500	PIGQ-001 (ENST00000321878)
PIGQ-10/11.2R	ttgacactgaggctgctcc		PIGQ-001 (ENST00000321878)
CSNK1G1-2F	acccatttcagaagctatgca	600	CSNK1G1-001 (ENST00000303052)
CSNK1G1-2R	gtcttgaaacacaccaaagct		CSNK1G1-001 (ENST00000303052)

CSNK1G1-3F	gctgttctagccttgctaga	331	CSNK1G1-001 (ENST00000303052)
CSNK1G1-3R	aaccccaaaccattagcagt		CSNK1G1-001 (ENST00000303052)
CSNK1G1-4F	ggtgatcttagccctcgttt	354	CSNK1G1-001 (ENST00000303052)
CSNK1G1-4R	tcccctgtcaactcaactcc		CSNK1G1-001 (ENST00000303052)
CSNK1G1-5F	tcccattgaagctgttgtgc	447	CSNK1G1-001 (ENST00000303052)
CSNK1G1-5R	tgccatcaagcctgtgagta		CSNK1G1-001 (ENST00000303052)
CSNK1G1-6F	tcagtgaagtgactcagggc	629	CSNK1G1-001 (ENST00000303052)
CSNK1G1-6R	tcaagtactcacactgctcaga		CSNK1G1-001 (ENST00000303052)
CSNK1G1-7F	ggcaaaggaagtacacatcgg	437	CSNK1G1-001 (ENST00000303052)
CSNK1G1-7R	ctcaagcaatcctcccacct		CSNK1G1-001 (ENST00000303052)
CSNK1G1-8F	atctcccatgtttaggccct	403	CSNK1G1-001 (ENST00000303052)
CSNK1G1-8R	agatgaaaggccctgctcat		CSNK1G1-001 (ENST00000303052)
CSNK1G1-9F	gcttccccacaactgctatg	273	CSNK1G1-001 (ENST00000303052)
CSNK1G1-9R	gctggttggagaaaggaggt		CSNK1G1-001 (ENST00000303052)
CSNK1G1-10F	tcgaaggtaagctactctcca	481	CSNK1G1-001 (ENST00000303052)
CSNK1G1-10R	cctgagggttgcttgaaagt		CSNK1G1-001 (ENST00000303052)
CSNK1G1-11F	cctgtttagcgaccctgtct	384	CSNK1G1-001 (ENST00000303052)
CSNK1G1-11R	tcttccccgaaatcacagct		CSNK1G1-001 (ENST00000303052)
CSNK1G1-12F	cactgtagatggtgggtggt	610	CSNK1G1-001 (ENST00000303052)
CSNK1G1-12R	ctccttctgcagacaaagcc		CSNK1G1-001 (ENST00000303052)
CSNK1G1-11bF*	tcacatcctaacccctgcag	330	CSNK1G1-201 (ENST00000303032
CSNK1G1-11bR*	ggaaagcaaaaggagggtcg		CSNK1G1-201 (ENST00000303032
CBL-1F	cttcacgccctgcttctct	383	CBL-001 (ENST00000264033)
CBL-1R	cttcaccagacccgtccc		CBL-001 (ENST00000264033)
CBL-2F	tgggcaatggggttatggat	454	CBL-001 (ENST00000264033)
CBL-2R	ccattcaggcagtcacaga		CBL-001 (ENST00000264033)
CBL-3F	gtaacatcctcaacagcgca	490	CBL-001 (ENST00000264033)
CBL-3R	accaaagccaggaaatacataca		CBL-001 (ENST00000264033)
CBL-4F	gtggctctccttcctttcct	293	CBL-001 (ENST00000264033)
CBL-4R	tcaccgaagtagcagtaacca		CBL-001 (ENST00000264033)
CBL-5F	gccctctgagttggttgtac	300	CBL-001 (ENST00000264033)
CBL-5R	cctattgcagaaccttggct		CBL-001 (ENST00000264033)
CBL-6F	ccttccaccgtaataccagc	250	CBL-001 (ENST00000264033)
CBL-6R	ttggacagcccctaagttcc		CBL-001 (ENST00000264033)
CBL-7F	tacttacaccacgttgccct	378	CBL-001 (ENST00000264033)
CBL-7R	aagcatctagtctgggtcct		CBL-001 (ENST00000264033)
CBL-8F	aggacccagactagatgctt	387	CBL-001 (ENST00000264033)
CBL-8R	ggccaccccttgtatcagta		CBL-001 (ENST00000264033)
CBL-9F	cctggcttttggggttaggt	395	CBL-001 (ENST00000264033)
CBL-9R	agtgttttacggctttagaagac		CBL-001 (ENST00000264033)
CBL-10F	tgaaagatgccatttcccca	246	CBL-001 (ENST00000264033)
CBL-10R	aaacccactagtacacagcaa		CBL-001 (ENST00000264033)
CBL-11F	gattctttgctgtgtactagtgg	491	CBL-001 (ENST00000264033)
CBL-11R	gcctggcccacacatatttc		CBL-001 (ENST00000264033)
CBL-12F	cagaggctcagctgtggtaa	279	CBL-001 (ENST00000264033)
CBL-12R	tcatttctacatggtgcagaga		CBL-001 (ENST00000264033)

CBL-13F	acttgtcttggtcagcagaaat	338	CBL-001 (ENST00000264033)
CBL-13R	agcaggtgaagggtgtcaat		CBL-001 (ENST00000264033)
CBL-14F	tggcaaaacgagaagatgaatc	300	CBL-001 (ENST00000264033)
CBL-14R	agtcccagctacagaagaattt		CBL-001 (ENST00000264033)
CBL-15F	gctgccccgtattgaaatgt	476	CBL-001 (ENST00000264033)
CBL-15R	agagccagagtttaaggaatgac		CBL-001 (ENST00000264033)
CBL-16F	aaacccagccttgtgactga	500	CBL-001 (ENST00000264033)
CBL-16R	ccttctaggtgccacttgag		CBL-001 (ENST00000264033)
