# Supplementary material for: Clinical whole-genome sequencing in severe early-onset epilepsy reveals new genes and improves molecular diagnosis
Source: Hum Mol Genet. 2014 Jan 25;23(12):3200–11. doi: 10.1093/hmg/ddu030 (PMC4030775; doi:10.1093/hmg/ddu030)
Supplement: Supplementary Data [file supp_ddu030_ddu030supp.doc]

**Supplementary Material**

**Table of Contents**

1. Supplementary Notes S1-4

S1. Extended clinical descriptions………………………………………………….1

S2. Other interesting candidate variants deemed to be benign……..3

S3. Assay for UPD mosaicism in Patient 2…..………………………………….4

S4. Results from trios 5 and 6.……………………..………………………………..5

2. WGS500 Consortium: names and affiliations of authors……………………………………7

3. Supplementary References……………………………………………………………………………….7

1. **Supplementary Notes**

**S1. Extended clinical descriptions**

**Patient 1 – *KCNQ2***

Patient 1 was born at 41 weeks gestation to non-consanguineous Caucasian parents. He was delivered by emergency C-section due to failure to progress. His birth weight was 3.63kg and he was not dysmorphic. On day 1 he began having cyanotic episodes and then more obvious seizures (up to 200 per day). His EEG on day 1 was very abnormal with a discontinuous pattern, 2-3 seconds of EEG attenuation interrupted by burst of irregular slow/fast and sharp waves, the bursts occurring, both synchronously and asynchronously. There were a few periods of more continuous EEG, focal sharp waves were seen, mainly over the left mid central to parietal cortex. On day 14, the predominantly sleep EEG was more abnormal, characterized by synchronous and asynchronous bursts of irregular slow waves, with spike transients interrupted by 1-2 seconds of EEG attenuation (almost burst-suppression), with multifocal spikes standing out during the periods of EEG attenuation. An MRI scan showed generalized mild reduction in white matter bulk with a thin corpus callosum, but was otherwise normal. A diagnosis of OS was made.

Trials of clonazepam, vigabatrin, folinic acid and pyridoxal phosphate in the first month of life were largely ineffective, so these were discontinued. The patient continued having more than 20 mainly tonic spasms daily, lasting 2-3 minutes. He was put on topiramate, diazepam, and nitrazepam but the fits continued. At age 5 months, the fits were less severe. They stopped by age 17 months. The quasi burst suppression pattern persisted until age 3 months, after which the EEG became more continuous in wake and sleep. The EEG at age 20 months was more stable, dominated by drug-induced fast activity, with no epileptiform discharges, but the last EEG at 4 years, though continuous, again showed multifocal sharp/spike wave discharges, maximal over the right mid temporal cortex, spreading in sleep to the right fronto-central areas. The patient had severe developmental delay. By age 4, he could sit with support, hold objects, indicate ‘yes or no’ by facial gestures, and showed some hand regard and environmental awareness. He is now age 5, and is on topiramate and nitrazepam. His head circumference has moved from the 75th to the 25th percentile over time. He is small with height/length falling from the 0.4th percentile, weight remaining at the 0.4th percentile.

**Patient 2 – *KCNT1***

Patient 2 was born at term after a normal pregnancy to nonconsanguineous Caucasian parents. His father’s maternal aunt and grandmother were reported to have had epilepsy, and a paternal cousin “possible epilepsy”. He had mild jaundice at birth which resolved spontaneously. His head circumference was 32cm and weight 2.66kg. He was admitted to hospital on day 14 with severe tonic-clonic seizures. His EEG was severely abnormal, consistent with OS, with bursts of synchronous and asynchronous irregular spike wave discharges, interrupted by 2-3 seconds of EEG attenuation, when multifocal discharges stood out with a posterior emphasis (Supplementary Fig. 2). Some of the irregular spike wave bursts were associated with head jerks. He had about 40 seizures per day, lasting about one minute. At this stage, his MRI was normal but. At age 8 months, an MRI showed delayed myelination but a structurally normal brain. He was admitted to hospital age 15 months with increased seizure activity, at which point he was noted to have severe developmental delay, hypertonia, brisk reflexes, ankle clonus, with microcephaly and a metopic synostosis. He had frequent tonic seizures lasting 30-40 seconds. He had a trial of phentyoin and carbamazepine, which were unhelpful, and so valproate was initiated. This helped the seizures but made the patient very irritable, so valproate was discontinued and he was treated with topiramate and clobezam, which proved helpful. Topirimate was stopped and replaced with Keppra after the patient developed a kidney stone. At the age of 21 months, his head circumference was less than the 0.4th percentile. From the age of four, he has continued frequent seizures, including tonic and tonic-clonic fits, as well as episodes of status. He is now age 5.

**Patient 3 – *SCN2A***

Patient 3 was born at 42 weeks’ gestation by forceps delivery after a normal pregnancy. Her parents were Caucasian and nonconsanguineous. She was not dysmorphic, her birth weight was 3.81kg and her head circumference 36cm. She started having tonic-clonic and absence seizures at 20 hours and was given phenobarbitone. Her CT and MRI scans were normal but her first EEG was very abnormal, with normal background but multifocal sharp waves especially during sleep, mainly centro-temporal but also over the posterior cortex, right more than the left. She had 4-6 tonic spasms daily and was trialled with biotin, pyridoxine, carbamazepine and phenobarbitone. An EEG at age 6 weeks was consistent with an early epileptic encephalopathy evolving into an Ohtahara pattern, and a diagnosis of OS was given. The patient was taken off carbamazepine, biotin, pyridoxine and folinic acid and put on pyridoxal phosphate and vigabatrin. She showed no response to the latter and was subsequently treated with valproate and clobazam, which seemed to improve her seizures. At age 8 months, she was having approximately three minor tonic episodes daily but had no more significant fits, and her EEG was more continuous, showing drug-induced fast frequencies, with sharp waves limited to both posterior cortex. An MRI at age 1 year showed cerebral atrophy with delayed myelination and hypomyelination. At age 16 months, the patient was having minor absence-like episodes occasionally. She has not had seizures since age 2.

The patient showed severe developmental delay. At age 10 months, she could smile, laugh and vocalize. At age 3, her development was improving: she could move her arms and attempt to stand. Her head circumference was in the 50th percentile. Now age 4, she still has significant developmental delay. She is not able to sit or stand upright, though she has reasonable head control. She is confined to a wheelchair, is dependent for all needs, and is hypertonic in all limbs. Her head circumference is at the 9th percentile, height and weight 25th percentile.

**Patient 4 – *PIGQ***

Patient 4 was born at 41 weeks to West African parents who reported that they were non-consanguineous. His birth weight was 3.83kg and head circumference 32.5cm (2nd percentile). At age 4 weeks, he started having cyanotic episodes with eye twitching, followed by brief stiffening of the upper body. He was first seen medically after a prolonged seizure at age 3 months, and put on diazepam, phenytoin, phenobarbitone and paraldehyde. His MRI was normal but his EEG showed burst-suppression pattern with bursts of high amplitude multifocal, irregular sharp and slow wave discharge, interrupted by 1-1.5 seconds of EEG flattening, in wake and drowsiness, when the posterior discharges were prominent, consistent with OS. A subtle focal seizure was recorded. He was put on phenobarbitone, vigabatrin, biotin and pyridoxine. The vigabatrin was stopped and valproate started, but this was ineffective, so it was replaced with topiramate, subsequently clonazepam was added. When seen at 9 months, he had no smile, no head control, poor vision, hyptonia and a head circumference at the 10th percentile. He had thick lips, thick alveolar margins, a narrow palate, and inverted nipples. An MRI showed delayed/limited myelination but no gross structural lesion. The patient had a gastrostomy inserted at age 12 months due to difficulty feeding. By 14 months, he was having 10 seizures per day (tonic, tonic-clonic and cyanotic spells, mostly 1-2 minutes duration), and his head circumference had fallen from 50th percentile at 3 months to below the 0.4th percentile. At 21 months he had recurrent vomiting and aspiration pneumonia, his fits continued and he also had some odd dystonic movements. He was profoundly developmentally delayed. An EEG at age two remained very abnormal but more continuous in wake and sleep over the right hemisphere, with very active multifocal sharp waves on the left side, the left discharge interrupted in sleep by brief periods of EEG attenuation over the left hemisphere. A left temporal focal seizure was recorded. The patient developed a respiratory infection and died age 2 years 4 months.

**Patient 5 – *CSNK1G1***

Patient 5 was born in Bangladesh to non-consanguineous parents, after a normal pregnancy. Her birth weight was 2.49kg. On day 2, she had a prolonged convulsion requiring admission to hospital for 10 days. A CT scan showed mild generalized “atrophy” of the brain. An EEG showed abnormal, polyrhythmic spikes and waves bilaterally. She was diagnosed with tonic/clonic epilepsy, having 4-5 seizures per day. Seizures were controlled with phenobarbitone, which was stopped at age 1 ½ years; subsequently the seizures recurred. She first presented in the UK at age 8 with severe epilepsy and developmental delay. She was still having 2-4 seizures per day, and an EEG showed a diffusely slow background activity, with multifocal high amplitude sharp waves in wake, the discharge followed by periods of EEG flattening when the child was relaxed. The conclusion was that the EEG showed a multifocal seizure potential on a background of significant disruption of cortical function. At that time she was treated with valproate. She was microcephalic, with a head circumference <3rd percentile. She had full lips, a broad nasal root, significant hypotonia but no other diagnostic or dysmorphic features. She was treated variously with clobazam, topiramate, phenobarbitone, Keppra and melatonin. Her developmental progress had been very slow; at age 8, she could clap hands and stand with support. She was still having frequent tonic/clonic seizures when last seen a year ago, and is now age 19.

**Patient 6 – *CBL***

Patient 6 was born in India to non-consanguineous parents, after a normal pregnancy. Her birth weight was 3kg. On day 25 she developed signs of heart failure and was treated with diuretics. She was found to have a patent ductus arteriosis (PDA) and an atrial septal defect (ASD). At age 2.5 months, she developed tonic seizures, having up to 6 per day. She was diagnosed in India as having had a “cerebral infarction”. A CT scan age 6 months showed “significant global volume loss with consequent extra axial effusions/hygromas”. Her seizures initially remitted but then recurred at age 18 months, and they have been very frequent since that time. She was first seen in the UK at age 6, at which point she was having up to six seizures per day. She had normal limb movement and reflexes, with no localizing neurological signs. Cranial MRI and angiography performed at that time showed no obvious sign of a cerebral infarct or vascular abnormality. She was noted to have extensive hypopigmented skin patches on her thorax, abdomen and arms. She had severe developmental delay and microcephaly (head circumference 41.2cm). Her EEG at age 6 was abnormal, the background diffusely of low amplitude, with multifocal sharp waves, most frequent over the midline fronto-central areas, appearing in drowsiness and sleep. She was treated with valproate, clobazam and Keppra. She had normal limb movement and reflexes.

**S2. Other interesting candidate variants deemed to be benign**

**Trio 2**

Patient 2 had a *de novo* 7kb deletion encompassing the promoter region and the first three exons of the transcription factor *SP3*. This was too small to have been detected by array, and since the coverage across the first three exons was low in all samples due to high GC content, it would likely have been missed by exome sequencing too. Sp3 regulates many genes involved in brain development[^1^](#_ENREF_1), so could conceivably be linked to epilepsy. However, the fact that heterozygous *SP3* knockout mice are phenotypically normal[^2^](#_ENREF_2), combined with the more compelling *KCNT1* finding, made this a less plausible candidate.

**Trio 4**

Genes encoding sodium and potassium channels are frequently mutated in epilepsy. Therefore, we first considered two compound heterozygous, nonsynonymous SNVs identified in *SCN4A*. One (D952N) was novel and the other (M1808I) had a frequency 0.002 in 1000 Genomes. The channel encoded by *SCN4A,* Nav1.4, is primarily known as a skeletal muscle channel, and mutations in it are known to cause various muscle diseases[^3^](#_ENREF_3). *SCN4A* is expressed in the brain (Allen Brain Atlas), so it seemed plausible that these compound heterozygous mutations could be responsible for the seizures in this patient, as well as his severe hypotonia. However, the patient’s unaffected sibling was also found to be a compound heterozygote (data not shown), so these *SCN4A* variants were deemed benign.

The second candidate mutation we considered in Patient 4 was a *de novo* nonsynonymous mutation at a highly conserved position in *C5ORF42*. Compound heterozygous mutations in this gene were recently reported to cause Joubert Syndrome (JS)[^4^](#_ENREF_4), a recessive disorder characterized by a distinct mid-hindbrain malformation, hypotonia, ocular-motor apraxia and hyperpnea. JS patients occasionally suffer from seizures[^5^](#_ENREF_5), but since our patient did not have the diagnostic “molar tooth sign” on the MRI or any breathing abnormalities, and we could not find a second mutation in *C5ORF42* that would suggest recessive inheritance, we concluded that this was not a case of misdiagnosis and that the mutation was not the cause of OS in this patient.

**Trio 6**

Patient 6 also had two compound heterozygous variants in *ANK3*, one novel (NM_001149:c.G541A:p.V181I) and the other seen in one other WGS500 sample (NM_020987:c.A9758T:p.E3253V). This gene encodes ankyrin-G, which plays an important role in anchoring ion channels at the axon initial segments and nodes of Ranvier[^6^](#_ENREF_6). A defect in ankyrin-G might disrupt ion channel placement and thus lead to misregulation of action potential initiation and propagation. However, we think it unlikely that the combination of these two heterozygous variants would strongly affect ankyrin-G function, since one of them, V181I, recapitulates the amino acid seen at this position in ankyrin-R and ankyrin–B, and this is a conservative amino acid substitution, so the variant is probably benign.

**S3. Assay for UPD mosaicism in Patient 2**

To confirm the uniparental isodisomy (UPD), DNA from Patient 2 (OTH_5) was run on the HumanCytoSNP-12v2.1 array (Illumina Inc). Only 1 out of 11,976 SNPs genotyped on chromosome 9 was called as heterozygous, conclusively validating the UPD and showing that it extended along the whole chromosome. Reviewing the single SNP that was called heterozygote (rs1407858) indicated that this gave a low signal due to another SNP nearby (rs78989195) that was homozygous for the non-reference allele (Supplementary Fig. 3a). By analyzing another 9 SNPs with intermediate B allele frequencies and/or low Log R ratios, we identified one SNP (rs2013762) that failed genotyping because it was situated inside a 2.7kb deletion (Supplementary Fig. 3a) that was homozygous in OTH_5, inherited from his heterozygous father (OTH_6). Although the deletion is not reported in the Database of Genomic Variants, its relatively small size likely precludes its detection by array-based methods. This deletion did not disrupt any genes and, as it was detected in 3 out of 198 other unrelated samples in WGS500 (frequency = 0.008), it was considered unlikely to be of clinical significance.

Although the B allele frequency along the chromosome 9 plot did not detect any obvious mosaicism, it is difficult to pick up chromosomal anomalies that are present in <5% of cells using such methods[^7^](#_ENREF_7). Therefore, we designed primers around the deletion flanking rs2013762 so we could conduct a PCR-based assay that would be more sensitive to low level mosaicism. Primers were designed adjacent to the deletion breakpoints using Primer3 (http://frodo.wi.mit.edu/). An additional reverse primer was designed inside the deletion such that it would only anneal to the non-deleted chromosome (Supplementary Table 5 and Supplementary Fig. 3). PCR amplification was carried out using 20ng of DNA template and the FastStart PCR kit (Roche). Bands of the expected sizes were obtained in both PCRs for the heterozygote father, whilst only the deletion-specific PCR worked for OTH_5, consistent with the absence of a maternally inherited chromosome. We repeated these PCRs using patient DNAs obtained from both peripheral blood and saliva, increasing the number of cycles from 35 to 40 (Supplementary Fig. 3b). The lack of product for the PCR specific for the non-deleted chromosome, even after these extra cycles, confirms that there is unlikely to be a subpopulation of cells that retain a maternally inherited chromosome.

To confirm the specificity of the deletion-specific PCR, the amplicon was purified using exonuclease I (NEB, Ipswich, MA) and Shrimp Alkaline Phosphatase (USB, Cleveland, OH) and then sequenced using BigDye chemistry (Applied Biosystems, Foster City, CA). The Sanger trace confirmed the deletion coordinates to be chr9:100,786,557-100,789,296 (GRCh37/hg19) (Supplementary Fig. 3c), consistent with the HighSeq200 data, with CCACCT microhomology seen at the deletion breakpoints. It is thought microhomology such as this can help mediate deletions through mechanisms such as Fork Stalling and Template Switching (FoSTeS)[^8^](#_ENREF_8).

UPD can be caused by a number of mechanisms, including monosomy rescue, post-fertilization error, trisomy rescue and gamete complementation. When isodisomy involves the whole chromosome, the most likely mechanisms are monosomy rescue and post-fertilization error[^9^](#_ENREF_9). Mosaicism is not often seen in the case of monosomy rescue, due to the lethality of the monosomic cell line, but it can usually be detected if the isodisomy results from post-fertilization error. In the case of OTH_5, the fact that we failed to detect the maternal chromosome even at low levels of mosaicism means that the most likely mechanism is monosomy rescue: a nullisomic oocyte was fertilized by a normal sperm, leading initially to a zygote with chromosome 9 monosomy. This would normally be embryonically lethal. However, an early non-disjunction event that duplicated the father’s chromosome rescued the embryo but also led to homozygosity of the pathogenic *KCNT1* variant. Given that two separate mutational events were involved, we consider that the recurrence risks in this family are extremely low.

**S4. Results from trios 5 and 6**

**Patient 5: *CSNK1G1***

We identified a *de novo* nonsynonymous mutation in *CSNK1G1*, encoding a casein kinase***.*** The mutation, NM_022048:c.C688T:p.R230W, disrupts a highly conserved residue within the catalytic domain that packs against the activation segment of the kinase. It is likely that the introduction of a large side chain such as that of tryptophan will affect activation segment dynamics and therefore activity. Casein kinase 1 is involved in synaptic transmission[^10^](#_ENREF_10) and also plays a key role in Wnt signalling[^11^](#_ENREF_11), which is crucial for multiple embryonic processes including neuronal differentiation[^12^](#_ENREF_12) and craniofacial development[^13^](#_ENREF_13). There is also evidence that *CSNK1G2* is associated with febrile seizures[^16^](#_ENREF_16). Thus, this mutation in *CSNK1G1* is a good candidate for causing the severe early-onset epilepsy in Patient 5.

**Patient 6: *CBL***

In Patient 6 we detected two *de novo* mutations in the *CBL* gene that were 93bp from each other. A single Illumina read suggested these were *in cis* and this was subsequently confirmed using allele-specific PCR and Sanger sequencing (data not shown). Recent work suggests that in eukaryotes, around 3% nucleotide substitutions are made up of such multi-nucleotide events[^17^](#_ENREF_17). Of the two mutations, one was synonymous (NM_005188:c.C1320T:p.G440G) and the other predicted to affect splicing (NM_005188:exon9:c.1228-1G>A) (Supplementary Fig. 1f). We extracted RNA from the patient’s blood and showed that the latter mutation leads to the skipping of exon 9 (Supplementary Fig. 5). Cbl ubiquitinates receptor tyrosine kinases that signal to the Ras/MAPK pathway[^18^](#_ENREF_18), prompting their degradation. Exon 9 encodes the C-terminal part of the RING finger domain that is responsible for Cbl’s ubiquitin ligase activity.

Cbl is primarily recognized as a tumour suppressor in haematological malignancies[^19^](#_ENREF_19). However, germline mutations in it and other genes in the Ras pathway also cause the neuro-cardio-facial-cutaneous (NCFC) syndromes[^20^](#_ENREF_20). Heterozygous missense mutations in the RING domain of *CBL* have been reported to cause facial, cutaneous and cardiac abnormalities, hypotonia and developmental delay[^21^](#_ENREF_21), as well as microcephaly and a predisposition to juvenile myelomonocytic leukemia (JMML). Mutations in *KRAS* and *BRAF* have been reported to cause cardio-facial-cutaneous syndrome (CFCS) with refractory epilepsy[^24^](#_ENREF_24). Our patient did not have the typical cutaneous symptoms of CFCS, but she did have a large hypo-pigmented patch and had been treated for an atrial septal defect as an infant, suggesting a “CFC-like” syndrome. However, she was not facially reminiscent of Noonan syndrome.

Curiously, Niemayer *et al.* reported two JMML patients with an almost identical splice site mutation to ours[^22^](#_ENREF_22). One patient had developmental delay and reduced growth but neither was reported to have epilepsy. Niemayer *et al*. found that their mutation (1228-2A>G) gave rise to some transcripts with premature stop codons, in addition to the one that we observed with the in-frame deletion of exon 9, due to 1228-1G>A (Δ exon 9). The retention of the LZ dimerization domain in the Δ exon 9, but not the 1228-2A>G mutant, may account for the different consequences, since one would anticipate that Δ exon 9 Cbl would retain its adaptor function in multiple cellular signalling pathways.

*CBL* mutations are notorious for their variable phenotypes and incomplete penetrance, even in the case of the recurrent Y371H mutation[^23^](#_ENREF_23). Thus, although the JMML patients with the 1228-2A>G mutation were not reported to have epilepsy, this *CBL* mutation still seems the primary candidate in Patient 6. Given that we did not find any other likely pathogenic *CBL* mutations in the larger panel of epilepsy patients, it may be that a) *CBL* mutations only rarely manifest themselves primarily as epilepsy, b) this mutation is not causal, or c) there are additional modifier variants in this patient.

**2. WGS500 Consortium: names and affiliations of authors**

**Steering Committee:** Peter Donnelly (Chair)^1^, John Bell^2^, David Bentley^3^, Gil McVean^1^, Peter Ratcliffe^1^, Jenny Taylor^1,4^, Andrew Wilkie^4,5^

**Operations Committee:** Peter Donnelly^1^ (Chair), John Broxholme^1^, David Buck^1^, Jean-Baptiste Cazier^1^, Richard Cornall^1^, Lorna Gregory^1^, Julian Knight^1^, Gerton Lunter^1^, Gil McVean^1^, Jenny Taylor^1,4^, Ian Tomlinson^1,4^, Andrew Wilkie^4,5^

**Sequencing & Experimental Follow up:** David Buck^1^ (Lead), Christopher Allan^1^, Moustafa Attar^1^, Angie Green^1^, Lorna Gregory^1^, Sean Humphray^3^, Zoya Kingsbury^3^, Sarah Lamble^1^, Lorne Lonie^1^, Alistair Pagnamenta^1^, Paolo Piazza^1^, Guadelupe Polanco^1^, Amy Trebes^1^

**Data Analysis:** Gil McVean^1^ (Lead), Peter Donnelly^1^, Jean-Baptiste Cazier^1^, John Broxholme^1^, Richard Copley^1^, Simon Fiddy^1^, Russell Grocock^3^, Edouard Hatton^1^, Chris Holmes^1^, Linda Hughes^1^, Peter Humburg^1^, Alexander Kanapin^1^, Stefano Lise^1^, Gerton Lunter^1^, Hilary Martin^1^, Lisa Murray^3^, Davis McCarthy^1^, Andy Rimmer^1^, Natasha Sahgal^1^, Ben Wright^1^, Chris Yau^6^

1. The Wellcome Trust Centre for Human Genetics, Roosevelt Drive, Oxford, OX3 7BN, UK

2. Office of the Regius Professor of Medicine, Richard Doll Building, Roosevelt Drive, Oxford, OX3 7LF, UK

3. Illumina Cambridge Ltd., Chesterford Research Park, Little Chesterford, Essex, CB10 1XL, UK

4. NIHR Oxford Biomedical Research Centre, Oxford, UK

5. Weatherall Institute of Molecular Medicine, John Radcliffe Hospital, Headington, Oxford OX3 9DS, UK

6. Imperial College London, South Kensington Campus, London, SW7 2AZ, UK

**3. References**

1. Li, L., He, S., Sun, J.M. & Davie, J.R. Gene regulation by Sp1 and Sp3. *Biochem Cell Biol* **82**, 460-71 (2004).

2. Kruger, I. *et al.* Sp1/Sp3 compound heterozygous mice are not viable: impaired erythropoiesis and severe placental defects. *Dev Dyn* **236**, 2235-44 (2007).

3. Raja Rayan, D.L. & Hanna, M.G. Skeletal muscle channelopathies: nondystrophic myotonias and periodic paralysis. *Curr Opin Neurol* **23**, 466-76 (2010).

4. Srour, M. *et al.* Mutations in C5ORF42 Cause Joubert Syndrome in the French Canadian Population. *Am J Hum Genet* **90**, 693-700 (2012).

5. Bachmann-Gagescu, R. *et al.* Genotype-phenotype correlation in CC2D2A-related Joubert syndrome reveals an association with ventriculomegaly and seizures. *J Med Genet* **49**, 126-37 (2012).

6. Dzhashiashvili, Y. *et al.* Nodes of Ranvier and axon initial segments are ankyrin G-dependent domains that assemble by distinct mechanisms. *J Cell Biol* **177**, 857-70 (2007).

7. Laurie, C.C. *et al.* Detectable clonal mosaicism from birth to old age and its relationship to cancer. *Nat Genet* **44**, 642-50 (2012).

8. Hastings, P.J., Ira, G. & Lupski, J.R. A microhomology-mediated break-induced replication model for the origin of human copy number variation. *PLoS Genet* **5**, e1000327 (2009).

9. Yamazawa, K., Ogata, T. & Ferguson-Smith, A.C. Uniparental disomy and human disease: an overview. *Am J Med Genet C Semin Med Genet* **154C**, 329-34 (2010).

10. Chergui, K., Svenningsson, P. & Greengard, P. Physiological role for casein kinase 1 in glutamatergic synaptic transmission. *J Neurosci* **25**, 6601-9 (2005).

11. Davidson, G. *et al.* Casein kinase 1 gamma couples Wnt receptor activation to cytoplasmic signal transduction. *Nature* **438**, 867-72 (2005).

12. Hirabayashi, Y. *et al.* The Wnt/beta-catenin pathway directs neuronal differentiation of cortical neural precursor cells. *Development* **131**, 2791-801 (2004).

13. Fossat, N., Jones, V., Garcia-Garcia, M.J. & Tam, P.P. Modulation of WNT signaling activity is key to the formation of the embryonic head. *Cell Cycle* **11**, 26-32 (2012).

14. Chan, D.W., Chan, C.Y., Yam, J.W., Ching, Y.P. & Ng, I.O. Prickle-1 negatively regulates Wnt/beta-catenin pathway by promoting Dishevelled ubiquitination/degradation in liver cancer. *Gastroenterology* **131**, 1218-27 (2006).

15. Bassuk, A.G. *et al.* A homozygous mutation in human PRICKLE1 causes an autosomal-recessive progressive myoclonus epilepsy-ataxia syndrome. *Am J Hum Genet* **83**, 572-81 (2008).

16. Yinan, M. *et al.* Polymorphisms of casein kinase I gamma 2 gene associated with simple febrile seizures in Chinese Han population. *Neurosci Lett* **368**, 2-6 (2004).

17. Schrider, D.R., Hourmozdi, J.N. & Hahn, M.W. Pervasive multinucleotide mutational events in eukaryotes. *Curr Biol* **21**, 1051-4 (2011).

18. Swaminathan, G. & Tsygankov, A.Y. The Cbl family proteins: ring leaders in regulation of cell signaling. *J Cell Physiol* **209**, 21-43 (2006).

19. Kales, S.C., Ryan, P.E., Nau, M.M. & Lipkowitz, S. Cbl and human myeloid neoplasms: the Cbl oncogene comes of age. *Cancer Res* **70**, 4789-94 (2010).

20. Denayer, E. & Legius, E. What's new in the neuro-cardio-facial-cutaneous syndromes? *Eur J Pediatr* **166**, 1091-8 (2007).

21. Martinelli, S. *et al.* Heterozygous germline mutations in the CBL tumor-suppressor gene cause a Noonan syndrome-like phenotype. *Am J Hum Genet* **87**, 250-7 (2010).

22. Niemeyer, C.M. *et al.* Germline CBL mutations cause developmental abnormalities and predispose to juvenile myelomonocytic leukemia. *Nat Genet* **42**, 794-800 (2010).

23. Perez, B. *et al.* Germline mutations of the CBL gene define a new genetic syndrome with predisposition to juvenile myelomonocytic leukaemia. *J Med Genet* **47**, 686-91 (2010).

24. Adachi, M., Abe, Y., Aoki, Y. & Matsubara, Y. Epilepsy in RAS/MAPK syndrome: two cases of cardio-facio-cutaneous syndrome with epileptic encephalopathy and a literature review. *Seizure* **21**, 55-60 (2012).
