# Supplementary material for: Clinical whole-genome sequencing in severe early-onset epilepsy reveals new genes and improves molecular diagnosis
Source: Hum Mol Genet. 2014 Jan 25;23(12):3200–11. doi: 10.1093/hmg/ddu030 (PMC4030775; doi:10.1093/hmg/ddu030)
Supplement: Supplementary Data [file supp_ddu030_ddu030supp_file.doc]

**Supplementary Figure 1:** Sanger validation for presumed causal variants. Sequencing was carried out in both directions, although only the coding direction is shown here.  (a) Trio 1: *KCNQ2* - chr20:62071051:G>A; (b) Trio 3: *SCN2A* - chr2:166245874:A>G; (c) Trio 2: *KCNT1 -* chr9:138675924:G>A; (d) Trio 4: *PIGQ -* chr16:625837:A>G; (e) Trio 5: *CSNK1G1 -* chr15:64499785:G>A; (f) Trio 6: *CBL* - chr11:119149219:G>A.

**Supplementary Figure 2:** Electroencephalogram for Patient 2 during wake (A) and sleep (B) at day 14, exhibiting a “burst-suppression” pattern. Note the bursts of synchronous and asynchronous irregular spike wave discharges, interrupted by 2-3 seconds of EEG attenuation. See Supplementary Note S1 for a full clinical description.

**Supplementary Figure 3:** Confirmation of UPD in Patient 2 (OTH_5). (a) Top panel shows B allele frequency and Log R Ratio plots from the CytoSNP12 array. This confirms that the whole chromosome is homozygous, despite normal copy number. Middle and lower panels show the SNP cluster plots and the Illumina sequence data viewed with the Integrated Genome Viewer, respectively. Bottom left panel: rs1487858 failed genotyping due to a nearby SNP. Bottom right panel: rs2013762 failed because it is inside a homozygous 3 kb deletion. (b) Gel picture showing PCR products specific for the deleted and non-deleted chromosomes. The father is heterozygous for the deletion, hence the presence of both bands, while the affected individual only has the band for the deleted chromosome. The lack of any band for the normal chromosome, even after 40 cycles, confirms that the patient does not have any detectable sub-population of cells that retain the maternal chromosome 9, consistent with monosomy rescue being the most likely mechanism. (c) Sanger sequencing confirmed the identity of the deletion-specific amplicon and showed the exact genomic coordinates to be chr9:100,786,557-100,789,296.

**Supplementary Figure 4:** The *PIGQ* splicing mutation causes skipping of exon 3. These Sanger traces show the results of sequencing the RT-PCR products (primer PIGQ-5R) in Figure 3. They confirm the exon boundary for the aberrant PIGQ transcript and demonstrate that the skipping of exon 3 in OTH_13 still leaves the reading frame intact.  The predicted protein would be expected to be missing 44 amino acids.

**Supplementary Figure 5:** The *de novo* mutation in Patient 6 (EOE_18) in *CBL* disrupts splicing. (a) The variant causes skipping of exon 9. This image shows the Bioanalyzer gel from an RT-PCR (see Methods) and demonstrates the presence of two *CBL* transcripts in the heterozygous child. The blue arrow indicates the band expected from the annotated transcript, and the red arrow that expected from the skipping of exon 9. We would expect the resultant protein to be missing 68 amino acids. (b) Sequencing RT-PCR products confirms exon boundary for aberrant CBL transcript. In EOE_18, the skipping of exon 9 leaves the reading frame intact.  Sequence shown is from primer CBL-8F.
