# Supplementary material for: Clinical whole-genome sequencing in severe early-onset epilepsy reveals new genes and improves molecular diagnosis
Source: Hum Mol Genet. 2014 Jan 25;23(12):3200–11. doi: 10.1093/hmg/ddu030 (PMC4030775; doi:10.1093/hmg/ddu030)
Supplement: Supplementary Data [file supp_ddu030_ddu030supp_tables.doc]

**Supplementary Table 1:** Breakdown of phenotypes in Australian epileptic encephalopathy cohort.

| **Syndrome** | **Number** |
| --- | --- |
| Atypical Benign Partial Epilepsy of Childhood (ABPEC) | 6 |
| Dravet Syndrome | 19 |
| Epileptic encephalopathy with Continuous Spike and Wave during Sleep (ECSWS) | 10 |
| Epileptic Encephalopathy | 172 |
| Early Myoclonic Encephalopathy (EME) | 5 |
| Early Onset Epileptic Encephalopathy (EOEE) (onset before age 3 months) | 38 |
| Epilepsy-Aphasia | 27 |
| Febrile Infection-Related Epilepsy Syndrome (FIRES) | 12 |
| Infantile Spasms (IS) | 85 |
| Landau-Kleffner Syndrome (LKS) | 3 |
| Lennox-Gastaut Syndrome (LGS) | 38 |
| Myoclonic Atonic Epilepsy | 81 |
| Ohtahara syndrome | 4 |
| **Total** | **500** |

**Supplementary Table 2:** Phenotypes of additional patients sequenced in UK cohort.

| **Case** | **Age at onset** | **Syndrome** | **Seizure Type** | **EEG** | **MRI** |
| --- | --- | --- | --- | --- | --- |
| 1 | Neonatal | Ohtahara | Tonic | Burst suppression | normal |
| 2 | 15 weeks | EME | Myoclonic/ tonic | Diffusely slow, no focal or generalised paroxysmal activity despite myoclonic jerks during recording | normal |
| 3 | Neonatal | Ohtahara | Tonic | Burst suppression | normal |
| 4 | Day 1 | Ohtahara | Tonic | Burst suppression | Incomplete myelination temporal poles, thin corpus callosum |
| 5 | Neonatal | Ohtahara | Tonic | Burst suppression | normal |
| 6 | Neonatal | Ohtahara | Tonic | Burst suppression | normal |
| 7 | 1.5 months | Ohtahara | Flexor, then tonic | Burst suppression | normal |
| 8 | Day 3 | Ohtahara | Tonic | Burst suppression, some multifocal discharges | Severe asymmetrical cortical & subcortical atrophy, low T2 signal in basal ganglia, high T2 signal in white matter & focal cortical areas associated with neuronal depletion on spectroscopy |
| 9 | Neonatal | Ohtahara | Tonic | Burst suppression | Not known |
| 10 | Neonatal | Ohtahara | Tonic | Burst suppression | Not known |
| 11 | Neonatal | Ohtahara | Tonic | Burst suppression | Not known |

**Supplementary Table 3** (in separate Excel spreadsheet)**:** Molecular inversion probes used for screening the genes in the large epileptic encephalopathy cohort.

**Supplementary Table 4** (in separate Excel spreadsheet)**:** Primers used for Sanger sequencing the genes in the UK Ohtahara cohort.

**Supplementary Table 5:** Primers used for PCR assay to test for UPD mosaicism in OTH_5.

| **Target** | **Forward Primer** | **Reverse Primer** | **Annealing temperature** | **PCR size** |
| --- | --- | --- | --- | --- |
| Normal chromosome | AAGGCTCTTATTTATGCAATTTCC | GCATAGCAGAGCACACAGGA | 55°C | 398bp |
| Deleted chromosome | *as above* | TCCTGGTTTCTTCCAACTGC | 55°C | 663bp* |

*The predicted 3,403 bp product from the normal chromosome (chr9:100,786,413-100,789,815) is too long to amplify under standard PCR conditions.
